# Genetic analysis of over half a million people characterises C-reactive protein loci

**DOI:** 10.1038/s41467-022-29650-5

**Published:** 2022-04-22

**Authors:** Saredo Said, Raha Pazoki, Ville Karhunen, Urmo Võsa, Symen Ligthart, Barbara Bodinier, Fotios Koskeridis, Paul Welsh, Behrooz Z. Alizadeh, Daniel I. Chasman, Naveed Sattar, Marc Chadeau-Hyam, Evangelos Evangelou, Marjo-Riitta Jarvelin, Paul Elliott, Ioanna Tzoulaki, Abbas Dehghan

**Affiliations:** 1 Department of Epidemiology and Biostatistics, School of Public Health, Imperial College London, London, UK; 2 Cardiovascular and Metabolic Research Group, Department of Life Sciences, Brunel University London, London, UK; 3 The Centre for Inflammation Research and Translational Medicine (CIRTM), Brunel University London, London, UK; 4 Centre for Health and Well-being Across the Life Course, Brunel University London, London, UK; 5 Centre for Life Course Health Research, University of Oulu, Oulu, Finland; 6 Research Unit of Mathematical Sciences, University of Oulu, Oulu, Finland; 7 Estonian Genome Centre, Institute of Genomics, University of Tartu, Tartu, Estonia; 8 Department of Intensive Care, University Hospital Antwerp, Antwerp, Belgium; 9 Department of Hygiene and Epidemiology, University of Ioannina Medical School, Ioannina, Greece; 10 Institute of Cardiovascular and Medical Sciences, University of Glasgow, Glasgow, G12 8TA UK; 11 Department of Epidemiology, University of Groningen and University Medical Centre Groningen, Groningen, the Netherlands; 12 Division of Preventive Medicine, Brigham & Women’s Hospital, Boston, MA 02115 USA; 13 Department of Medicine, Brigham and Women’s Hospital, Harvard Medical School, Boston, MA USA; 14 MRC-PHE Centre for Environment and Health, School of Public Health, Imperial College London, London, W2 1PG UK; 15 UK Dementia Research Institute at Imperial College London, Burlington Danes Building, Hammersmith Hospital, DuCane Road, London, W12 0NN UK; 16 National Institute for Health Research Imperial Biomedical Research Centre, Imperial College London, London, W2 1PG UK

**Keywords:** Data processing, Chronic inflammation, Genome-wide association studies

## Abstract

Chronic low-grade inflammation is linked to a multitude of chronic diseases. We report the largest genome-wide association study (GWAS) on C-reactive protein (CRP), a marker of systemic inflammation, in UK Biobank participants (N = 427,367, European descent) and the Cohorts for Heart and Aging Research in Genomic Epidemiology (CHARGE) Consortium (total N = 575,531 European descent). We identify 266 independent loci, of which 211 are not previously reported. Gene-set analysis highlighted 42 gene sets associated with CRP levels (*p* ≤ 3.2 ×10^−6^) and tissue expression analysis indicated a strong association of CRP related genes with liver and whole blood gene expression. Phenome-wide association study identified 27 clinical outcomes associated with genetically determined CRP and subsequent Mendelian randomisation analyses supported a causal association with schizophrenia, chronic airway obstruction and prostate cancer. Our findings identified genetic loci and functional properties of chronic low-grade inflammation and provided evidence for causal associations with a range of diseases.

## Abstract (web summary)

Inflammation is associated with a variety of diseases. Here, the authors identify 266 genetic loci associated with C-reactive protein levels, a marker of inflammation, in >500,000 Europeans, along with associated pathways, clinical outcomes and potential causal associations with disease.

## Introduction

Chronic inflammation is the prolonged inflammatory response to stimulating agents, injury or dysregulated acute inflammation^1^. Chronic low-grade inflammation is associated with numerous complex disorders including; several cancers, cardiovascular disease (CVD), respiratory disease, autoimmune diseases and endocrine-metabolic conditions^2–7^. However, the potential molecular pathways linking chronic low-grade inflammation with chronic diseases are poorly understood.

C-reactive protein (CRP), an acute phase protein predominantly produced by the liver^8–11^, has been widely studied as a marker of systemic inflammation. Environmental and genetic factors contribute substantially to serum CRP levels. Previous genetic association studies have identified 58 distinctive loci explaining ~7% of the variation of CRP levels using data from ~200,000 Europeans^12^. Still, the genetic architecture of this complex trait is not well characterised. Unravelling the underlying genetic components of circulating CRP levels can elucidate mechanisms of involvement of CRP in disease processes and highlight potential therapeutic targets for modulating inflammation.

Here, we report the largest genome-wide association study (GWAS) on CRP levels, using data from the UK Biobank (UKB) and the Cohorts for Heart and Aging Research in Genomic Epidemiology (CHARGE) consortia^12^. We conducted an array of post-GWAS analyses to elucidate the functional characteristics of the findings and highlight potential underlying pathways. Lastly, we perform a phenome-wide association study (PheWAS) to agnostically investigate clinical consequences of chronic inflammation and complement that with Mendelian Randomisation (MR) analyses to assess causal relations.

## Results

### Genetic loci associated with CRP levels in UKB

**Fig. 1:**
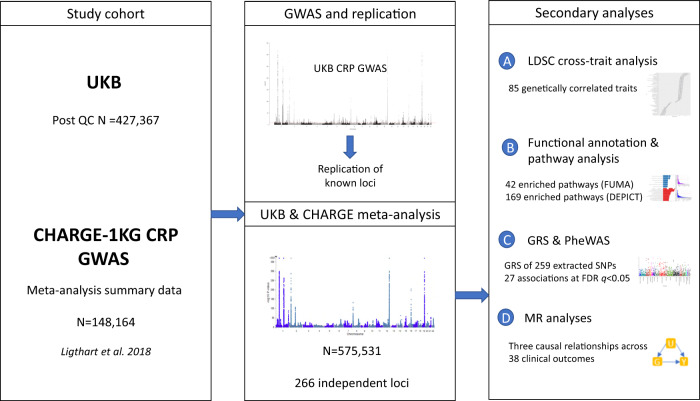
Schematic overview of the study. UKB = UK BioBank, QC = quality control, 1KG = 1000 genomes, CHARGE = Cohorts for Heart and Aging Research in Genomic Epidemiology consortia, LDSC = LD score regression, FUMA = Functional Mapping and Annotation of GWAS, DEPICT = data-driven Expression Prioritised Integration for Complex Traits, GRS = genetic risk score, MR = Mendelian randomisation.

The study design is illustrated in Fig. 1, and the detailed characteristics of the subjects, exclusion criteria and phenotype source are described in Supplementary Tables 1–4. After exclusions (“methods”), 427,367 UKB participants contributed to the GWAS analysis which identified 49,164 SNPs associated with CRP levels (at genome wide significance (GWS) of *p* < 5 × 10^−8^). Out of these, we mapped 293 independent loci by using the Functional Mapping and Annotation of GWAS (FUMA)^13^ platform. The variance explained by these independent variants within the UKB GWAS loci was 16.3%. We replicated all 57 previously reported loci^12^ (HLA region excluded) (Supplementary Table 5).

### UKB and CHARGE GWAS meta-analysis

**Fig. 2:**
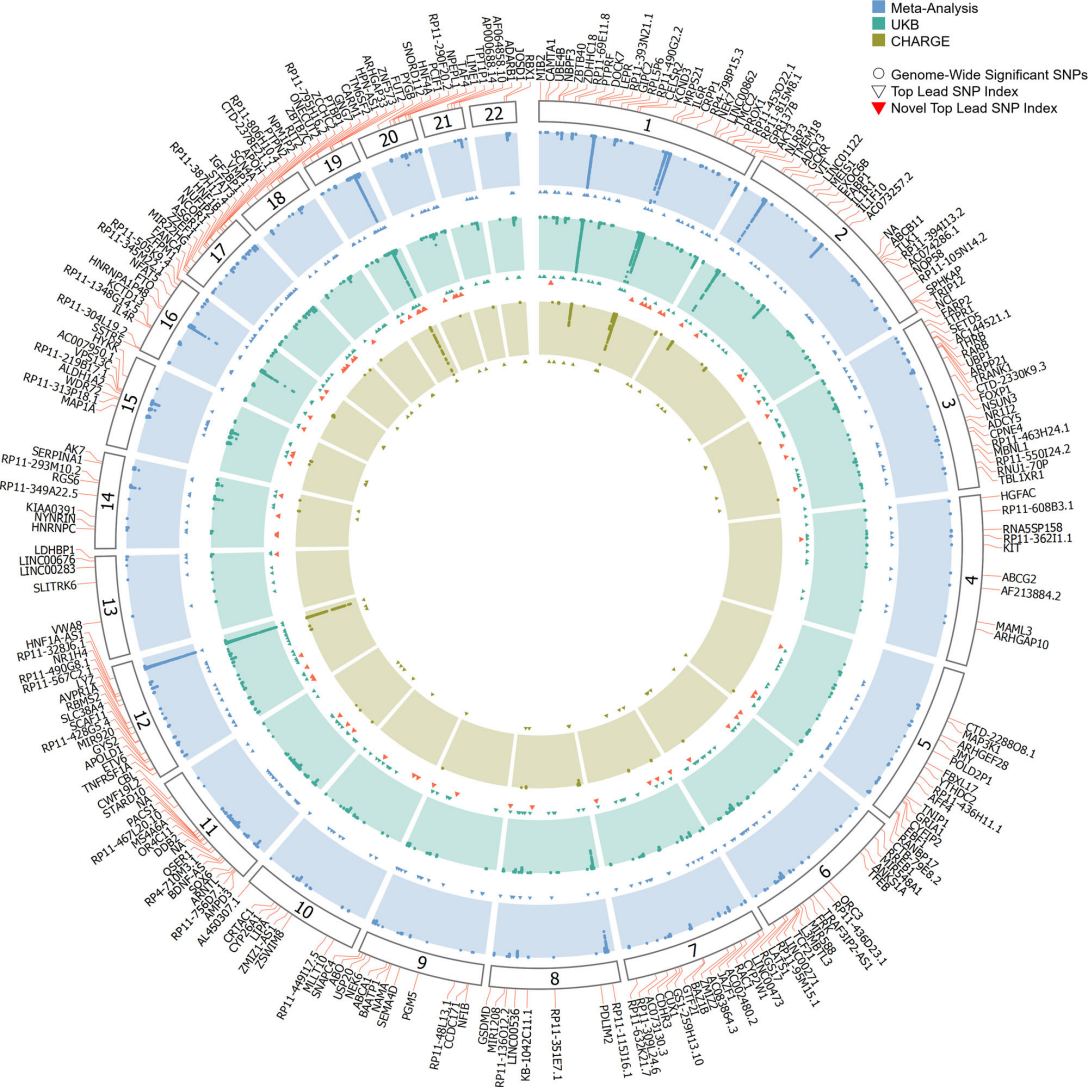
Circle Manhattan plot. Genome wide significant hits at *p* < 5 × 10^−8^ are presented for CHARGE CRP meta-GWAS (inner circle), UKB CRP GWAS (middle circle) and meta-analysis of UKB-CHARGE (outer circle). Labelled genes are the sentinel SNPs of each 266 loci nearest genes.

We meta-analysed UKB GWAS results with summary statistics from published CHARGE GWAS meta-analysis and identified 48,912 genetic variants associated with CRP at GWS level (Fig. 2). The LDSC intercept was 1.15 (SE = 0.02) in UKB GWAS, consequently, genomic control was applied. A second genomic control was applied to the meta-analysis result reducing the intercept to one and LDSC ratio < 0. The GWS SNPs mapped to 266 distinct loci (Supplementary Data 1), 211 have not been previously reported, and 55 are previously reported loci. The top three not previously identified loci associated with CRP included, rs11868378 at the *RP11-806H10.4* locus (*β* = −0.033, *p* < 5.74 × 10^−34^), rs55707100 at the *MAP1A* locus (*β* = 0.069, *p* < 5.79 × 10^−28^) and rs6073958 within the *PCIF1;PLTP* locus (*β* = 0.028, *p* = 5.87 × 10^−28^) (Table 1). The UKB-CHARGE CRP meta-analysis results were used for all subsequent downstream analyses.

### Credible set analysis of CRP associated loci

Fine mapping for likely causal variants within the CRP associated genomic loci identified 95% credible sets of variants (the smallest number of variants which posterior probability sum to at least 95% probability) using a Bayesian framework. There was 91 (34%) loci with <10 variants within the 95% credible set. In 23 (9%) loci the 95% credible set comprised of one variant and 12 (5%) other loci included two variants. The top three loci with the largest number of variants within the 95% credible set were *LATS1;KATNA1* with 362, *RANBP17* with 288 and *PYGB* with 279 variants (Supplementary Data 2).

### Functional annotation and pathway enrichment

**Fig. 3:**
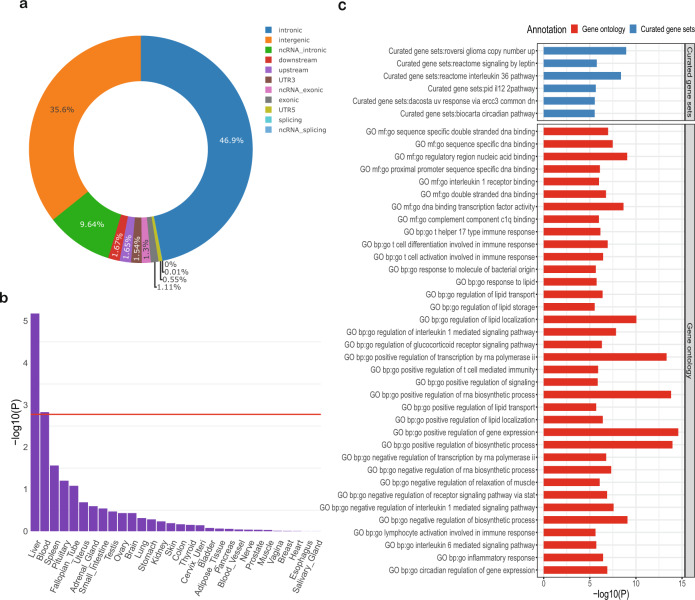
Functional analysis of CRP based on functional annotation and MAGMA gene-property analysis. **a** Functional annotation of the variants in the genomic risk loci of the CRP meta-analysis by ANNOVAR plotted by the proportion of annotated SNPs (independent significant). **b** MAGMA gene-property analysis results are shown for average expression of 30 general tissue types, the red line indicates the Bonferroni threshold (*p* = 1.67 × 10^−3^). **c** MAGMA gene-set analysis plot of the Bonferroni significant (*p* < 3.23 × 10^−6^) gene-sets.

We applied a range of functional annotation analyses to leverage the CRP GWAS results using FUMA-MAGMA and DEPICT. Our FUMA ANNOVAR results found that 82.5% of significant SNPs (*p* < 5 × 10^−8^) and SNPs in LD with the significant SNPs are located within intronic and intergenic regions (Fig. 3a). MAGMA gene-based analysis annotated SNPs to 19,122 protein coding genes, of which there were 1475 genes associated with CRP at Bonferroni significance (*p* ≤ 2.61 × 10^−6^) (Supplementary Fig. 1, Supplementary Data 3). The top five genes from the gene-based analysis were *NECTIN2* (alias *PVRL2*) (*p* = 8.40 × 10^−162^), *PDE4B* (*p* = 3.90 × 10^−159^), *OASL* (*p* = 1.49 × 10^−154^), *IL6R* (*p* = 1.16 × 10^−148^) and *APOE* (*p* = 5.32 × 10^−147^). In total, the gene mapping results from FUMA (consisting of positional mapping, eQTL mapping and chromatin interaction mapping) and MAGMA gene-based analysis had a combined 1062 unique mapped genes demonstrating that CRP levels are associated with an overarching range of functional genes (Supplementary Data 4).

We conducted gene-set analysis using MAGMA and DEPICT. MAGMA tested 15,478 gene sets, and prioritised 42 after Bonferroni correction (*p* ≤ 3.23 × 10^−6^) and 255 at false discovery rate (FDR) < 0.05 (Fig. 3b and Supplementary Table 6). The prioritised gene-sets are involved in regulation of DNA expression, metabolites or immune and inflammatory response (Supplementary Table 6). DEPICT tested 10,968 gene sets, prioritised 169 gene-sets at Bonferroni significance (*p* ≤ 0.05/10,968 = 4.56 × 10^−6^) and 1387 at FDR < 0.05 (Supplementary Data 5). Further clustering identified 138 groups of gene sets which correlated and clustered in three sets, the larger two clusters mainly consisting of immune and DNA regulation pathways and the smaller cluster of metabolic pathways (Supplementary Fig. 2). When we combined the FUMA and DEPICT pathway analysis full summary results, 478 matched, of which, nuclear receptor transcription pathway, recycling of bile acids and salts, and cytokine signalling in immune systems were FDR significant in both. Some gene-sets of interest from the pathway enrichment include circadian pathway (*p* = 3 × 10^−6^, Supplementary Table 6), haemopoietic or lymphoid organ development, hemopoieses, extramedullary haematopoiesis and abnormal haematopoiesis (*p* = 7.35 × 10^−8^, 9.80 × 10^−8^, 4.39 × 10^−9^, 6.24 × 10^−5^ respectively, Supplementary Data 5).

We found that the prioritised genes were enriched for expression in the liver (*p* = 3.04 × 10^−6^) and whole blood (*p* = 4.24 × 10^−4^) using MAGMA and in precursor cells B lymphoid (*p* = 8.21 × 10^−7^), synovial fluid (*p* = 1.46 × 10^−5^), liver (*p* = 2.11 × 10^−5^) and blood (*p* = 2.66 × 10^−5^) using DEPICT (Supplementary Tables 7–8, Fig. 3b,c and Supplementary Fig. 3).

### Analysis of genetic relationships between CRP and other traits and diseases

**Fig. 4:**
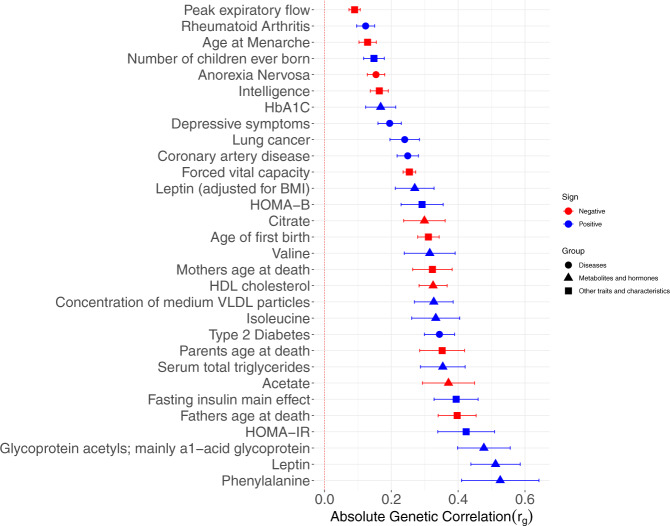
Cross-trait genetic correlation of traits with CRP. Ordered by group and ascending *p* value. The error bars correspond to the SE.

SNP-based heritability estimate for CRP in the UKB-CHARGE meta-analysis was 13%. We identified significant genetic correlation between CRP and 85 traits (*p* ≤ 0.05/192 = 2.6 × 10^−4^), though, many of the traits were related (Supplementary Data 6). We found moderate genetic correlations (r_g_~0.5) for four phenotypes, including leptin, phenylalanine, triglycerides in small high-density lipoprotein-HDL, and glycoproteins. Figure 4 depicts the unique Bonferroni significant traits in three broad groups including metabolites, chronic/complex diseases, and risk factors.

**Fig. 5:**
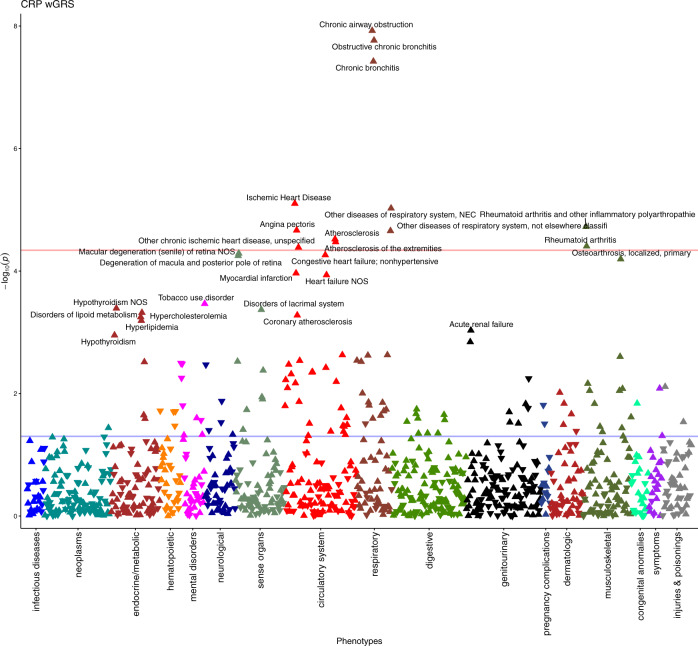
CRP weighted GRS PheWAS Manhattan plot. The red line indicates the Bonferroni threshold (*p* < 4.47 × 10^−5^) and the blue line indicates the nominal threshold (*p* < 0.05). The triangle pointing up represents positive association and down a negative association. All FDR significant phenotypes are annotated (FDR *q* < 0.05).

Using PheWAS, a weighted genetic risk score (GRS) based on UKB-CHARGE meta-analysis results was associated with 27 different outcomes at FDR < 0.05, of which, 12 were also Bonferroni significant (*p* < 0.05/1,118 = 4.47 × 10^−5^) (Fig. 5, Supplementary Table 9). We identified no phenome-wide significant clinical outcomes associated with a weighted GRS based only on cis-acting SNPs at *CRP* gene (Supplementary Fig. 4).

We assessed the causal role of genetically raised CRP on outcomes that were significant in PheWAS (*n* = 27) or were investigated in recent studies^14^ (*n* = 11). Results are interpreted as per 1 standard deviation (SD) increase in genetically raised natural log CRP levels. The 27 PheWAS clinical outcomes were initially assessed using MR outcome estimates obtained from the UKB PheWAS (MR-UKB). Then was assessed using MR outcome estimates obtained from published GWAS summary statistics with non-UKB sample populations for the available PheWAS identified outcomes (MR-rep). Firstly, MR -UKB using UKB-PheWAS result for SNP-outcome identified 17 (out of 27) IVW Bonferroni significant outcomes, six displayed consistent effect direction across all methods of which, degeneration of macular and posterior pole of retina and macular degeneration (senile) of retina sensitivity test had *p* < 0.05 (Supplementary Data 7). To replicate our findings, we conducted MR-rep (Supplementary Data 8, Supplementary Fig. 5). Of the 22 tested outcomes (since GWAS summary data were not identified for five of the 27 clinical outcomes), chronic obstructive pulmonary disease (COPD) (chronic airway obstruction) had a Bonferroni significant IVW MR estimate (*β* = 0.330, *p* = 7.94 × 10^−4^). Clinical outcome that reached nominal significance with consistent effect direction but did not have IVW significant estimates in MR-UKB were, hyperlipidaemia (*β* = 0.323, *p* = 0.008) and disorders of lipoprotein metabolism (*β* = 0.14, *p* = 0.042).

**Fig. 6:**
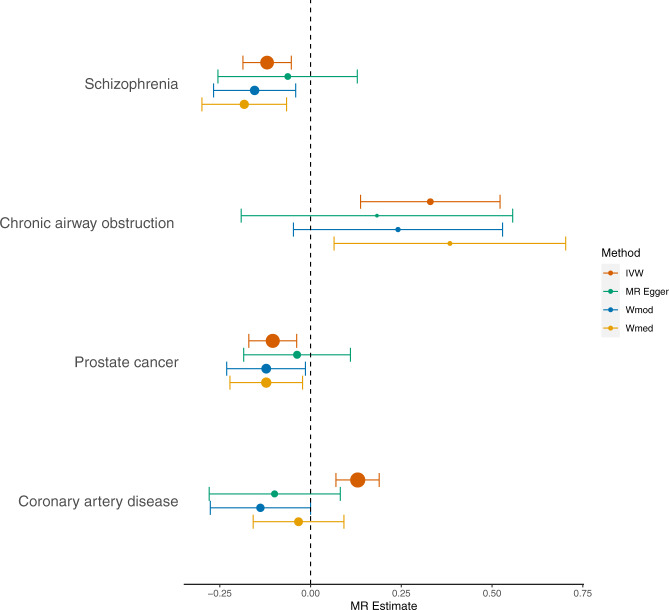
Two-sample Mendelian Randomisation results. Schizophrenia, chronic airway obstruction and prostate cancer survived Bonferroni threshold with consistent effect direction across sensitivity tests, many of which are also nominally significant. Coronary artery disease is presented here as a disease of interest. The size of the point represents the precision of the estimate (1/SE). The points are the beta estimates from the MR analyses and the error bars are the 95% confidence intervals.

To assess further outcomes that are of interest to chronic inflammation but may have been underpowered in PheWAS we conducted Two-sample MR analyses using published GWAS’s (Supplementary Table 4). We used CRP associated sentinel variants as genetic instruments (trans-CRP IVs) in the MR analyses and conducted sensitivity analyses with variants at the CRP locus (cis-CRP IVs) (Supplementary Fig. 6, Supplementary Data 9–10). MR IVW analysis confirmed that genetically elevated CRP levels (per 1-unit difference in natural log-transformed CRP) are associated with a reduced risk of schizophrenia (*β* = −0.120, *p* = 4.14 × 10^−4^), with consistent results across sensitivity tests. A positive association of genetically elevated CRP levels on breast cancer was identified with IVW (*β* = 0.061, *p* = 3.56 × 10^−3^), with concordant direction of effect across MR methods. Major depressive disorder (MDD) had a positive IVW association close to the Bonferroni threshold (*β* = 0.069; *p* = 5.27 × 10^−3^) with concordant sensitivity tests. MR-PRESSO identified at least one outlying variant for all outcomes except MDD and stroke. However, the exclusion of the pleiotropic variant did not notably affect the result. The analyses using cis-acting CRP IVs which survived the Bonferroni threshold was prostate cancer (*β* = −0.104, *p* = 0.002). Outcomes at nominal significance included; schizophrenia (*β* = −0.130, *p* = 0.005), type 1 diabetes (*β* = 0.274, *p* = 0.015) and autism spectrum disorder (*β* = 0.118, *p* = 0.045). The Bonferroni significant MR results with supported sensitivity analyses are displayed in Fig. 6. Lastly, bidirectional MR did not provide evidence for reverse causality between schizophrenia, breast cancer, prostate cancer and COPD (as exposures) and CRP levels (as outcome) (Supplementary Table 10).

## Discussion

Taking advantage of data from > 500,000 individuals, we have expanded the number of genomic loci associated with circulating CRP levels from 58 to 266 and have improved the percentage of variance explained from ~7%^12^ to 16.3%. Further, our GWAS replicated 57 loci that were previously reported to associate with CRP^12,15–17^. Moreover, we report 85 traits genetically correlated with serum CRP and highlight 42 biological pathways underpinning CRP regulation. Through MR analysis we were able to provide evidence for a causal effect of low-grade chronic inflammation as measured by genetically elevated serum CRP on lower risk of schizophrenia and prostate cancer, and a higher risk of COPD.

In observational studies, CRP concentrations have an inverse linear relationship with pulmonary function^18^, and a positive association with COPD and mortality in COPD patients^19,20^. COPD is characterised by chronic inflammation^21^. Smoking is a major causal factor for COPD which induces an inflammatory response driven by CRP, IL-6 and TNF-alpha and persists even after smoking cessation^22^. However, raised CRP levels are also reported in COPD patients independent of smoking status, proposing CRP as a marker of systemic inflammation that occurs in these patients^23,24^. Using PheWAS analysis in the UKB, we identified a potentially causal association between genetically elevated CRP and risk of chronic airway obstruction. This finding was validated with subsequent two-sample MR analyses using data from GWAS consortia. Previously, analyses by Daul et al. were too underpowered (with partial *r*^2^ of CRP instrument from 0.4 to 1.8%) to find an association between genetic variants in *CRP* gene and COPD^25^. Our results suggest the dysregulated chronic low-grade inflammation may mediate development of COPD or its progression.

The MR analyses is in agreement with prior reports on the protective causal role of increased CRP levels on the risk of schizophrenia^12,26–28^. Observational studies have reported a higher risk of schizophrenia with higher circulatory levels of CRP in younger age^29^, which suggest a possible role of acquiring infections in younger age in the development of schizophrenia later in life. In addition, neonatal studies have shown low levels of acute phase proteins such as CRP relate to increased risk^30^ and development^31^ of schizophrenia^28^. Although the exact underlying mechanism is not known, one possibility is that a genetic profile for stronger immune responses (i.e. higher genetic score for inflammation), may lead to a lower chance for infection in childhood, which is thought to be related to the risk of schizophrenia in adulthood.

We identified an inverse potential causal association between genetically elevated CRP levels and risk of prostate cancer. Observational studies have shown increased circulating CRP levels associated with the increased risk of prostate cancer^32^; however, the causality has not been established^33,34^. One may speculate that an inflammatory response inhibits early stages of oncogenesis for example by complement factor activation, which is regulated by CRP, promotes cancer cell death^35^. A genetically strong inflammatory response may play a proactive role against prostate cancer over the course of life.

Our study highlights haematopoiesis association with elevated CRP, which has not been previously reported^36^. The pathway analyses highlighted haematopoiesis pathways and tissue enrichment analyses highlighted whole blood, haematopoietic system, lymphoid progenitor/precursor cells and bone marrow cells. This demonstrates the identified genetic paths for CRP production affect the maturation of blood cells via series of different CRP related mapped genes, such as *CSF2*, *TNF* (alias *TNF-alpha*) and *IL1*^37,38^. Also, CRP regulation and haematopoietic development may share pathways potentially through cytokines such as IL1 and TNF-alpha^39,40^, yet, these results need a detailed examination for their basic and clinical meanings.

The strength of our study includes the large sample size of the UKB and the inclusion of CHARGE summary data which allowed us to replicate the findings and substantially extend the discovery panel. Several limitations are worth mentioning. Our discovery panel mainly consisted of participants of European ancestry; caution is needed when extending the findings to other ethnic groups. Although BMI influences chronic low-grade inflammation, it was not adjusted for in the study as previous GWAS^12^ addressed this and saw the vast majority of variants associate with CRP levels independently of BMI, there was also the concern of introducing collider bias^41,42^ in the following MR analyses. We did not investigate rare variants (MAF < 0.01) in our GWAS.

In conclusion, this large-scale effort more than tripled the number of known loci associated with CRP levels and provide a comprehensive picture of the genetic architecture of chronic inflammation. The loci provided insights into the biology of serum CRP through functional annotation and pathway analysis, such as the possible role of CRP in haematopoiesis. Finally, support for potential causality of low-grade chronic inflammation marked by CRP on risk of schizophrenia, chronic airway obstruction and prostate cancer highlights avenues of disease prevention through modulation of inflammation.

## Methods

Our research complies with all relevant ethical regulations. The UKB has ethics approval from the North West Multi-Centre Research Ethics Committee (11/NW/0382). Ethical approval was covered by the UKB and informed consent was obtained from participants. Data for this work was obtained under approved data request application ID 13436. Additional ethical approval was not required for the present study.

### Study design and study sample

The study design is depicted in Fig. 1. We used data from participants of European descent in UKB to conduct GWAS. Further, we performed one-stage meta-analysis using our UKB GWAS result and summary statistics from a GWAS meta-analysis conducted by the CHARGE consortium using 1000 Genomes imputed data from 49 studies (see Supplementary Table 1 presented the baseline characteristics for UKB participants. Baseline characteristics for the studies that contributed to the CHARGE meta-analysis have been described elsewhere^12^). The genomic positions used throughout this study was human genome assembly GRCh37 (hg19) from Genome Reference Consortium.

### GWAS on CRP levels in UKB population

We performed Linear Mixed Model (LMM) regression using BOLT-LMM version 2.3^43^ on CRP levels in UKB. This model accounts for cryptic relatedness within the sample. We used an additive genetic model, for all 8.9 million measured and imputed genetic variants. The model was adjusted for age, sex, UKB array (UKB vs UK BiLEVE to account for the different genotyping chips^44^) and 40 genetic principal components.

Serum CRP levels (mg/l was measured by immunoturbidimetry- a high sensitivity method on a Beckman Coulter AU5800 (ISO 17025:2005 accredited)^45^. CRP levels were transformed using natural log and the resulting range included was from −2.53 to 4.38, excluding individuals with extreme values ±4 SD from the mean. Individuals on immune modulating drugs, with auto-immune related diseases/disorders, which constituted 1.8% of the sample, were removed (Supplementary Table 2). We filtered variants with minor allele frequency (MAF) < 0.01 and imputation quality <0.1. The variance explained was calculated for the variants within the lead loci of the CRP UKB GWAS results using the formula^12^ [Eq. 1]:

1 $$\sum \;[(2\,\times \,{{{{{{\rm{MAF}}}}}}}_{{{{{{\rm{i}}}}}}}(1-{{{{{{\rm{MAF}}}}}}}_{i})\;{{B}_{i}}^{2})/{{{{\mathrm{var}}}}}({{{{\mathrm{ln}}}}}\,{{{{{\rm{CRP}}}}}})]$$

Where *∑* is the sum, *MAF*_*i*_ is the MAF of associated variant *i*, *ß*_*i*_ is the absolute effect estimate of the corresponding variant *i* on natural log CRP and *var* is the variance of natural log CRP levels obtained from AIRWAVE study^46^ (project AH-INT-052).

### Replication of previously reported sentinel SNPs

We looked up loci previously reported in CRP GWAS^12^ in our UKB GWAS. For every locus, SNP with smallest *p* value in the former GWAS was examined as a representative of that locus. We considered the finding as replicated when the sentinel SNP has a *p* < 0.01; and a concordant effect direction (Fig. 1).

### UKB and CHARGE GWAS meta-analysis

We conducted fixed-effects inverse variance-weighted meta-analysis of UKB GWAS summary statistics (*N* = 427,367) and CHARGE GWAS summary statistics (*N* = 148,164) using METAL^47^. Variants from the human leukocyte antigen (HLA) region (chr6: 25Mb-35Mb, hg19) in both UKB and CHARGE GWAS were excluded, as SNPs from the HLA region can lead to inflated test statistics and have been associated with multiple immunological traits^48^. Genomic control was applied to the UKB GWAS summary statistics prior to meta-analysis, while genomic control was already applied to CHARGE study, and then a final genomic control was applied to the meta-analysis results using the linkage disequilibrium (LD) score (LDSC) calculated genomic inflation factor. We determined independent genomic risk loci using Functional Mapping and Annotation of GWAS (FUMA)^13^ online platform (https://fuma.ctglab.nl/). FUMA clumps genome wide significant SNPs (*p* < 5 × 10^−8^) at specified *r*^2^ threshold to identify the independent and lead SNPs. In this instance we set *r*^2^ to 0.1 for independent and lead SNP definitions, making the number of SNPs identical. Independent associated SNPs residing in distinct LD blocks that physically overlap within a 500 kb window were merged into one locus. The sentinel SNP of each locus is the independent SNP with the smallest *p* value. The LD structures were based on the 1000 Genomes Project Phase 3 reference panel^49^ on European reference population and PLINK (v1.9^50^) was used to compute the *r*^2^.

### Credible set analysis

Fine-mapping was carried out on the CRP associated genomic loci to identify likely causal variants. Credible set analysis^51,52^ using the Bayesian framework was implemented. The posterior probability for variants to be causal was obtained by calculating the Bayes factors, which was then used to generate the 95% credible sets. The resulting variants of each loci are the smallest list of variants which cumulatively have a $\ge$ 95% probability of including causal variants.

### LD score regression

To provide a more accurate estimate of genetic inflation compared with effects attributable to true polygenicity and calculate SNP heritability, we applied LDSC regression using the LD-hub tool^53^. The genomic inflation factor obtained from the LDSC regression was used to correct for genomic inflation of the GWAS. LDSC analysis performs regression of GWAS meta-analysis summary statistics (using *χ*^2^ statistics) on the LD scores across the genome. When an LDSC intercept equals to one, this suggests no evidence of confounding bias, and an intercept larger than one suggests cryptic relatedness or population stratification as contributors to the genomic inflation reported. The proportion of inflation of the mean *χ*^2^ that the LDSC intercept ascribes to potential causes other than polygenic heritability is measured by the ratio (intercept−1/(mean χ^2^)−1)^54^. We utilised the European 1000 Genomes reference panel-based LD score file available in LD-hub.

To determine the genetic correlation of CRP with other phenotypic outcomes, we performed cross-trait LDSC analysis using publicly available GWAS summary statistics^54^ against the UKB-CHARGE CRP meta-analysis. In brief, the genetic covariance between two traits (e.g. CRP, LDL) is estimated by regressing the product of the *z*-score from the two studies against the LD-score, the slope of which is then multiplied by the number of tested SNPs^55^.

### Functional downstream analysis

To conduct in silico downstream functional analysis of the UKB-CHARGE CRP meta-analysis results, we used FUMA^13^, Multi-marker Analysis of GenoMic Annotation (MAGMA v1.6)^56^ and Data-driven Expression Prioritised Integration for Complex Traits (DEPICT)^57^. First, we performed functional annotation with FUMA of all genome-wide significant SNPs and SNPs in LD with them (*r*^2^ ≥ 0.6) using Annotate Variation (ANNOVAR) enrichment test (gene-based annotation), which annotates the functional consequence of SNPs on Ensemble (v92) protein coding genes (e.g. intron and exon)^58^. Functionally annotated SNPs were subsequently mapped to genes using three strategies: positional mapping (physical distance), expression quantitative trait loci (eQTL) mapping (eQTL association) and chromatin interaction described further in supplementary information. Furthermore, MAGMA was used to perform gene-based, gene-set and gene-property (tissue gene expression) analysis of the full GWAS meta-analysis summary results. In brief, gene-based analysis computes gene-based *p* values association statistics for SNPs that are mapped to protein coding genes. The gene-based *p* values are then used to compute gene-set *p* values in gene-set analysis. The SNPs mapped genes are tested for statistical overrepresentation in the predefined gene-sets. Gene-property analysis was conducted using eQTL gene expression data to identify tissue specificity of CRP. Multiple testing was corrected by using Bonferroni correction for gene-based and tissue-expression, and FDR for gene-set MAGMA analysis. In addition, DEPICT^57^ was conducted and its results were compared to FUMA results from MAGMA gene-based analysis and gene-property tissue enrichment analysis. DEPICT was used to predict gene functions to prioritise the most likely causal genes at associated loci, identify enriched pathways and specific tissues/cells where genes of the associated loci are expressed. The methods used for the downstream pathway analysis are described further in supplementary information.

### PheWAS

To explore effects of chronic inflammation as measured by CRP levels, we conducted PheWAS^59^ with subsequent MR analyses to asses causality of identified phenotypes. The phenotypic (including patient hospital records, cancer registry data, and death registry data defined as ICD codes from electronic medical records) and genotypic data (259 CRP associated sentinel SNPs) were extracted from the UKB database. Using the PheWAS ﻿(version 0.99.5-3) R package^60^, a total of 1118 hierarchical phecodes were directly matched to the ICD-9/10 codes, after filtering phecodes with <200 cases^61^, and patients that had similar or overlapping phenotypes from the corresponding control group were excluded. The minimum code count for a recorded event to be considered a case was one. We excluded non-White and related participants, adjusted for age, sex, BMI, and the first 15 principal components in the PheWAS logistic regression analyses. The genotypic data was constructed as a GRS for assessment in PheWAS by the summation of CRP-increasing alleles for each SNP, weighted by the beta coefficients of the SNP on circulating CRP levels from our meta-analyses. The weighted GRS was standardised by subtracting the GRS from the mean then divided by the SD. As the phenotypes are not completely independent in the phecode system, we utilised FDR (*q* < 0.05) as the overall determinant of significance accounting for multiple tests. A subsequent PheWAS was run utilising individual SNP genotypes and a subset of FDR significant phenotypes identified in the initial PheWAS to obtain individual estimates for MR. To assess pleiotropy of the *CRP* gene locus (±50 kb), using the same method above we calculated the weighted GRS of 29 independent (clumping window of 10,000 kb and an *r*^2^ threshold of 0.1) CRP SNPs and ran PheWAS.

### MR analyses

We applied two-sample MR analyses to assess the causal role of CRP on two sets of clinical outcomes: (1). The 27 clinical outcomes highlighted in PheWAS (FDR significant). For this set of outcomes, we initially used data from UKB (MR-UKB) (significance threshold *p* < 0.05/27 = 0.0019). Later we tried to replicate these results by using summary statistics form the largest published GWAS (MR-rep), using 245 trans-acting genetic variants (summary statistics available for 22 of 27 outcomes, significance threshold *p* < 0.05/22 = 0.0023). The published GWAS used are shown in Supplementary Table 3. (2) The 11 clinical outcomes that were suggested by the literature to be causally affected by CRP, using cis- and trans-acting CRP genetic variants (significance threshold *p* < 0.05/11 = 0.0045). Details of published GWAS used are shown in Supplementary Table 4.

We used fixed-effects inverse-variance weighted (IVW) MR^62^ as the main MR analysis method. Since IVW method is susceptible to heterogeneity, we conducted additional sensitivity MR analyses. Random-effects IVW (IVW-RE) MR was used as it allows heterogeneity in the estimates from individual genetic variants^63,64^. Sensitivity MR methods including weighted mode (W-mod), weighted median (W-med) and MR-Egger were used to investigate the degree of horizontal pleiotropy, a key violation to IV assumptions. MR-Egger allows an additional test for directional pleiotropy, with an assumption of INstrument Strengths being Independent of Direct Effects (InSIDE)^65^. Weighted median (W-med) MR gives a consistent causal estimate if at least half of the weight for the analysis comes from variants that are valid instruments^66^. Weighted mode (W-mod) MR provides a consistent estimate of the causal effect if a weighted plurality of the genetic variants are valid instruments^67^. Finally, MR-PRESSO can detect horizontal pleiotropy, test level of distortion between causal estimates from IVW and outlier corrected (*p*_distortion < 0.05); outlier-corrected IVW estimates are obtained by excluding pleiotropic variants from the analysis. This method requires that at least 50% of the variants are valid instruments and that the InSIDE assumption holds^68^.

SNPs that were in the vicinity of the *CRP* gene (50 kb downstream *APCS*-nearest gene upstream of *CRP* and 50 kb downstream of *CRP* was selected to capture lead variants in *CRP* locus) were selected as cis-acting variants (cis-CRP associated IVs) for sensitivity analysis in MR minimising the possibility of horizontal pleiotropy. After extracting the summary statistics for each outcome, effect estimates were aligned to have the same effective allele for exposure and outcome and were then clumped in a window of 10,000 kb and an *r*^2^ threshold of 0.1.

We assessed evidence of reverse causality for the reported outcomes with Bonferroni significant IVW MR and consistent sensitivity test results. Firstly, the exposure IVs for schizophrenia, breast cancer, prostate cancer and COPD were obtained from published GWAS summary statistics available through TwoSampleMR R package (Two-sample MR ID: ieu-b-42, ieu-a-1126, ieu-b-85, finn-a-J10_COPD, respectively). COPD exposure variants were selected at lowered *p* value threshold 1 × 10^−5^ due to low number of genome wide significant SNPs. Our CRP summary statistics was used for the association of IVs with CRP and was harmonised to the reported effect allele of the exposures. To assess reverse causality MR IVW and sensitivity methods were applied as described above.

### Reporting summary

Further information on research design is available in the Nature Research Reporting Summary linked to this article.

## Supplementary information

Supplementary Information

Description of Additional Supplementary Files

Supplementary Data 1

Supplementary Data 2

Supplementary Data 3

Supplementary Data 4

Supplementary Data 5

Supplementary Data 6

Supplementary Data 7

Supplementary Data 8

Supplementary Data 9

Supplementary Data 10

Reporting Summary

## Data Availability

The summary statistics of the CHARGE CRP GWAS used in this study is publicly available from the IEU open GWAS project accession code ieu-b-35 (Trait: C-Reactive protein level - IEU Open GWAS project (mrcieu.ac.uk)). The derived CRP GWAS meta-analysis summary statistics generated in this study have been deposited in the GWAS catalogue under accession code GCST90029070 (https://www.ebi.ac.uk/gwas/studies/GCST90029070). Human genome assembly GRCh37 (hg19) from Genome Reference Consortium https://www.sanger.ac.uk/data/genome-reference-consortium/).
