# Author Correction: Genetic analysis of over half a million people characterises C-reactive protein loci

**DOI:** 10.1038/s41467-022-31706-5

**Published:** 2022-07-05

**Authors:** Saredo Said, Raha Pazoki, Ville Karhunen, Urmo Võsa, Symen Ligthart, Barbara Bodinier, Fotios Koskeridis, Paul Welsh, Behrooz Z. Alizadeh, Daniel I. Chasman, Naveed Sattar, Marc Chadeau-Hyam, Evangelos Evangelou, Marjo-Riitta Jarvelin, Paul Elliott, Ioanna Tzoulaki, Abbas Dehghan

**Affiliations:** 1 Department of Epidemiology and Biostatistics, School of Public Health, Imperial College London, London, UK; 2 Cardiovascular and Metabolic Research Group, Department of Life Sciences, Brunel University London, London, UK; 3 The Centre for Inflammation Research and Translational Medicine (CIRTM), Brunel University London, London, UK; 4 Centre for Health and Well-being Across the Life Course, Brunel University London, London, UK; 5 Centre for Life Course Health Research, University of Oulu, Oulu, Finland; 6 Research Unit of Mathematical Sciences, University of Oulu, Oulu, Finland; 7 Estonian Genome Centre, Institute of Genomics, University of Tartu, Tartu, Estonia; 8 Department of Intensive Care, University Hospital Antwerp, Antwerp, Belgium; 9 Department of Hygiene and Epidemiology, University of Ioannina Medical School, Ioannina, Greece; 10 Institute of Cardiovascular and Medical Sciences, University of Glasgow, Glasgow, G12 8TA UK; 11 Department of Epidemiology, University of Groningen and University Medical Centre Groningen, Groningen, the Netherlands; 12 Division of Preventive Medicine, Brigham & Women’s Hospital, Boston, MA 02115 USA; 13 Department of Medicine, Brigham and Women’s Hospital, Harvard Medical School, Boston, MA USA; 14 MRC-PHE Centre for Environment and Health, School of Public Health, Imperial College London, London, W2 1PG UK; 15 UK Dementia Research Institute at Imperial College London, Burlington Danes Building, Hammersmith Hospital, DuCane Road, London, W12 0NN UK; 16 National Institute for Health Research Imperial Biomedical Research Centre, Imperial College London, London, W2 1PG UK

**Keywords:** Data processing, Chronic inflammation, Genome-wide association studies

Correction to: *Nature Communications* 10.1038/s41467-022-29650-5, published online 22 April 2022.

The original version of this article contained an error in the section “Data Availability”, which incorrectly read ‘The derived CRP GWAS meta-analysis summary statistics generated in this study has been deposited in the GWAS catalogue under accession code GCST00186 (https://www.ebi.ac.uk/gwas/)’. The correct version states ‘GCST90029070’ in place of ‘GCST00186’ and states “have” in place of “has”. This has been corrected in both the PDF and HTML versions of the Article.

