# Supplementary material for: Genetic analysis of over half a million people characterises C-reactive protein loci
Source: Nat Commun. 2022 Apr 22;13:2198. doi: 10.1038/s41467-022-29650-5 (PMC9033829; doi:10.1038/s41467-022-29650-5)
Supplement: Supplementary file 1 — Supplementary Information [file 41467_2022_29650_MOESM1_ESM.pdf]

# Genetic analysis of over half a million people characterises C-reactive protein loci

**Supplementary Methods**

**Supplementary Figures 1-6**

**Supplementary Tables 1-10**

**Supplementary References**

## Supplementary Methods

### *FUMA reference panel*

To define independent significant SNPs and genomic risk loci, FUMA uses the 1000G reference panel (including five population groups) and the UK Biobank release 2b reference panel (includes SNPs imputed with UK10K/1000G, included two population groups). Variants which do not exist in the selected reference panel are not included in any analyses[1]. More detail can be found elsewhere https://fuma.ctglab.nl/tutorial#refpanel .

### *Gene mapping in FUMA*

Functionally annotated SNPs were subsequently mapped to protein coding genes using three strategies to obtain prioritised genes associated to CRP in FUMA[1]:

1) positional mapping based on annotations obtained from ANNOVAR[2]. Physical distance of SNP from known protein coding genes was set to the default window of 10kb, in the human reference assembly (hg19/GRCH37).

2) eQTL mapping was used to map independent significant SNP and SNPs in LD of them to genes that show a significant association to cis-eQTLs (i.e. the allelic variation of the SNP association to with expression levels of the gene). This maps SNPs to genes up to 1MB apart. eQTL mapping used data repositories on all tissue types available in FUMA at the time, consisting of GTEx[3] v6-8, BRAINEAC[4], ComminMind Consortium[5], xQTL server[6], MuTHER[7], Blood eQTLs[8], eQTL Gen[9] , DICE[10], van der Wijst et al. scRNA eQTLs[11], PsychENCODE[12]. We used significant eQTL associations were FDR < 0.05 recommended in FUMA[1].

3) Chromatin interaction mapping is performed by overlapping independent significant SNPs and SNPs in LD of them with one end of significantly interacting regions in tissue/cell types. These SNPs are subsequently mapped to genes which promoter regions (by default 250 bp up-stream and 500 bp down-stream of transcription start site) overlap with another end of the significant interactions[1]. This mapping can involve long range interaction as there is no distance boundary. FUMA uses data on the

3D structure of chromatin interactions of 23 tissues and cell types in Hi-C[13] data, tissue and cell type from FANTOM[14–16] and PsychENCODE[12] The significance threshold was defined as FDR < 1 x $10^{-6}$ in FUMA[1] based on prior recommendations[13].

### *Gene based analysis*

MAGMA[17] using 1000 genomes phase 3 reference panels. For genome wide gene-based association analysis (GWGA) in MAGMA, input SNPs were mapped to protein coding genes obtained from Ensemble build 92, using a SNP-wise mode which analyses individual SNPs in a gene and aggregates the resulting SNP p-values into a gene test-statistic[17]. Genome-wide significance was determined by Bonferroni correction applied to 19,144 genes tested ($p < 2.61 \times 10^{-6}$).

### *Gene-set analysis*

MAGMA gene-set analysis is performed for curated gene sets and gene ontology (GO) terms obtained from molecular signature database (MsigDB) v5.2. This is to test whether the prioritised genes of the CRP GWAS meta-analysis clustered in specific biological pathways, cellular components or molecular functions. Competitive gene-set analysis examines if genes within the gene set are more strongly associated with each of the phenotypes than other genes. We performed the analysis with default parameters [1] using the results of the gene-based analysis. A total of 15,478 gene sets were tested, significance was determined by Bonferroni threshold ($p < 3.23 \times 10^{-6}$) to account for multiple testing burden[18].

### *Gene-property analysis*

To identify tissue specificity of CRP, the relationship between tissue specific gene expression profiles and CRP-gene associations was tested using MAGMA gene-property analysis[17] in FUMA[1]. This tested relationships between tissue specific gene expression profiles and CRP-gene associations based on the earlier computed gene-based p values. The tissue enrichment tested 30 broad tissue types and 53 specific tissues. Tissue types were obtained from GTEx[3] v7 RNA-sequence data, expression values were

log2 transformed average RPKM per tissue type. Multiple testing for 53 specific and 30 general tissue type was accounted by Bonferroni correction ($p < 9.43 \times 10^{-4}$, $p < 1.67 \times 10^{-3}$).

### *DEPICT*

DEPICT[19] *v1rel194* beta version for 1000G imputed data was used to identify enriched pathways, processed, tissue types and prioritised genes from each GWAS locus. This method uses the assumption that trait-relevant genes from independent GWAS loci from the same GWAS should often be functionally similar and therefore share functional annotations. Briefly, DEPICT uses 1000G p1v3 reference panel (CEU, GBR and TSI subpopulations, release date 2010-11-23 [20]) to perform LD-clumping for all input SNPs using plink v1.9[21] using r2 <0.1 and 1Mb window. Positions in the genome were defined according to genome build GRCh37/hg19. Precomputed DEPICT loci were defined as LD-blocks of $r^2>0.5$ (1000G pilot phase) from each independent SNP, HLA region (chr6: 25Mb-35Mb, genome build GRCh37/hg19) was omitted from analyses. This method uses reconstituted gene sets (are a consequence of the gene function prediction, which is quantified using co-regulation between gene pairs based o,, n gene expression data) derived from gene expression array data from ~80,000 samples and correlates their reconstituted gene set membership scores to obtain enrichment score. Empirical enrichment p-values were calculated by using the permutations over 500 randomly selected sets of gene-density-matched loci and subsequently FDR was calculated by performing 50 repetitions of scoring and adjustment steps. Further details of DEPICT analyses can be found elsewhere[19].

## Supplementary Figures

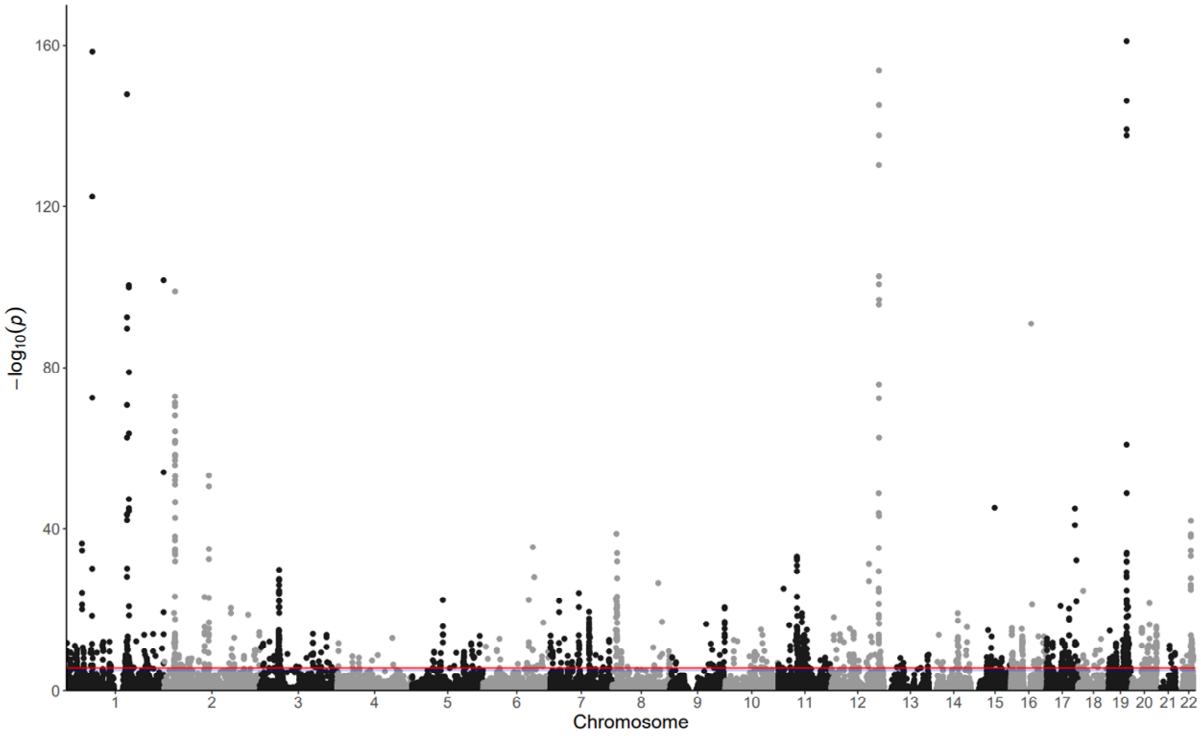


Supplementary Figure 1. Manhattan plot of genes association results for gene-based analysis of CRP.

The red line indicates genome wide significance (*p*= 2.62 x $10^{-6}$).

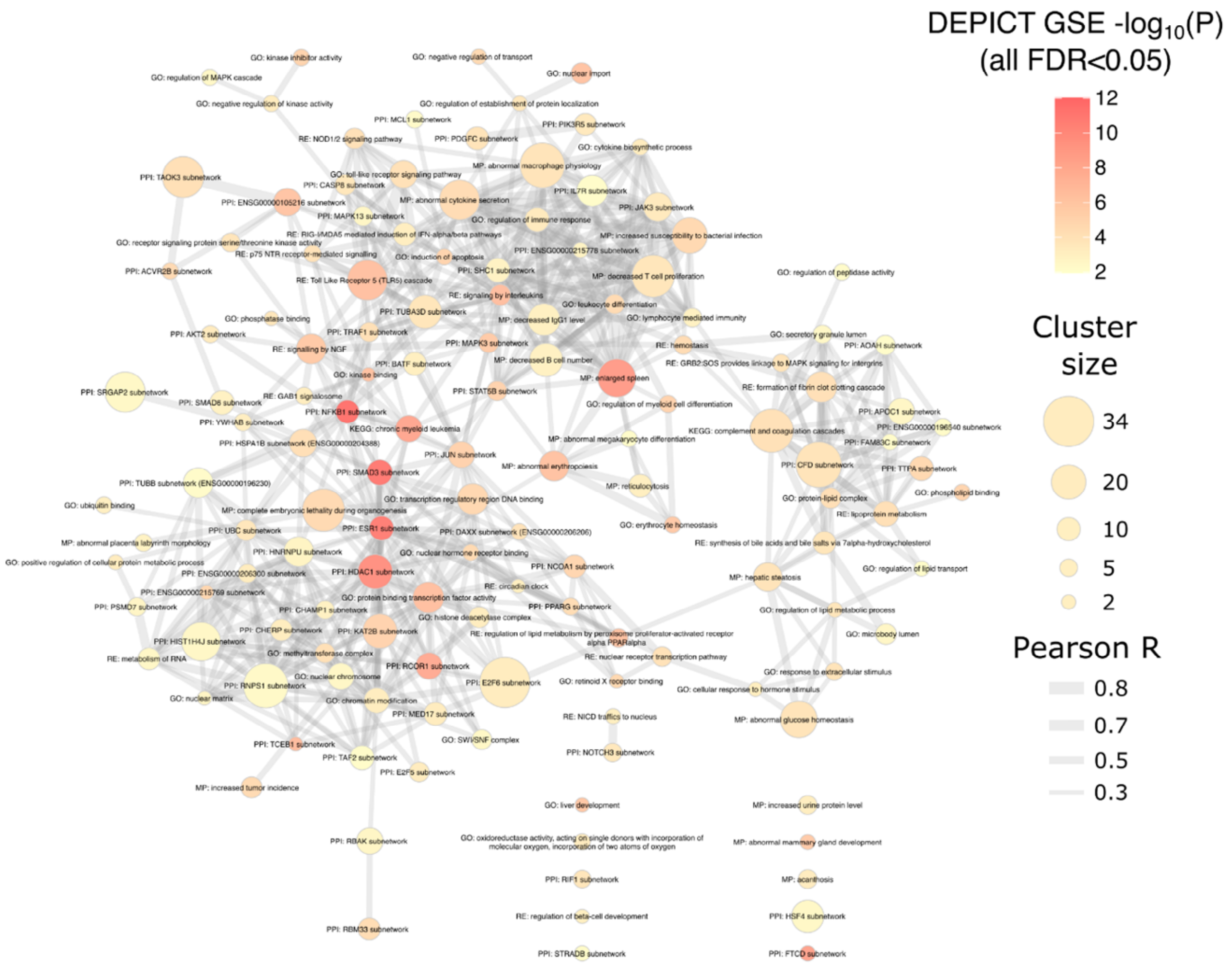


Supplementary Figure 2. Result of DEPICT gene set enrichment analysis. Each node represents an exemplar gene set from affinity-propagation clustering. There are 138 FDR <0.05 clusters of gene sets. Gene sets are represented by nodes coloured according to statistical significance, and links between them are indicated by their correlation (only correlations with $r > 0.3$ are shown). The size of the cluster is indicated by the size of the node. DEPICT GSE $-\log_{10}$ (P) refers to the gene-set enrichment two-sided p-value for that DEPICT gene-set generated by DEPICT.

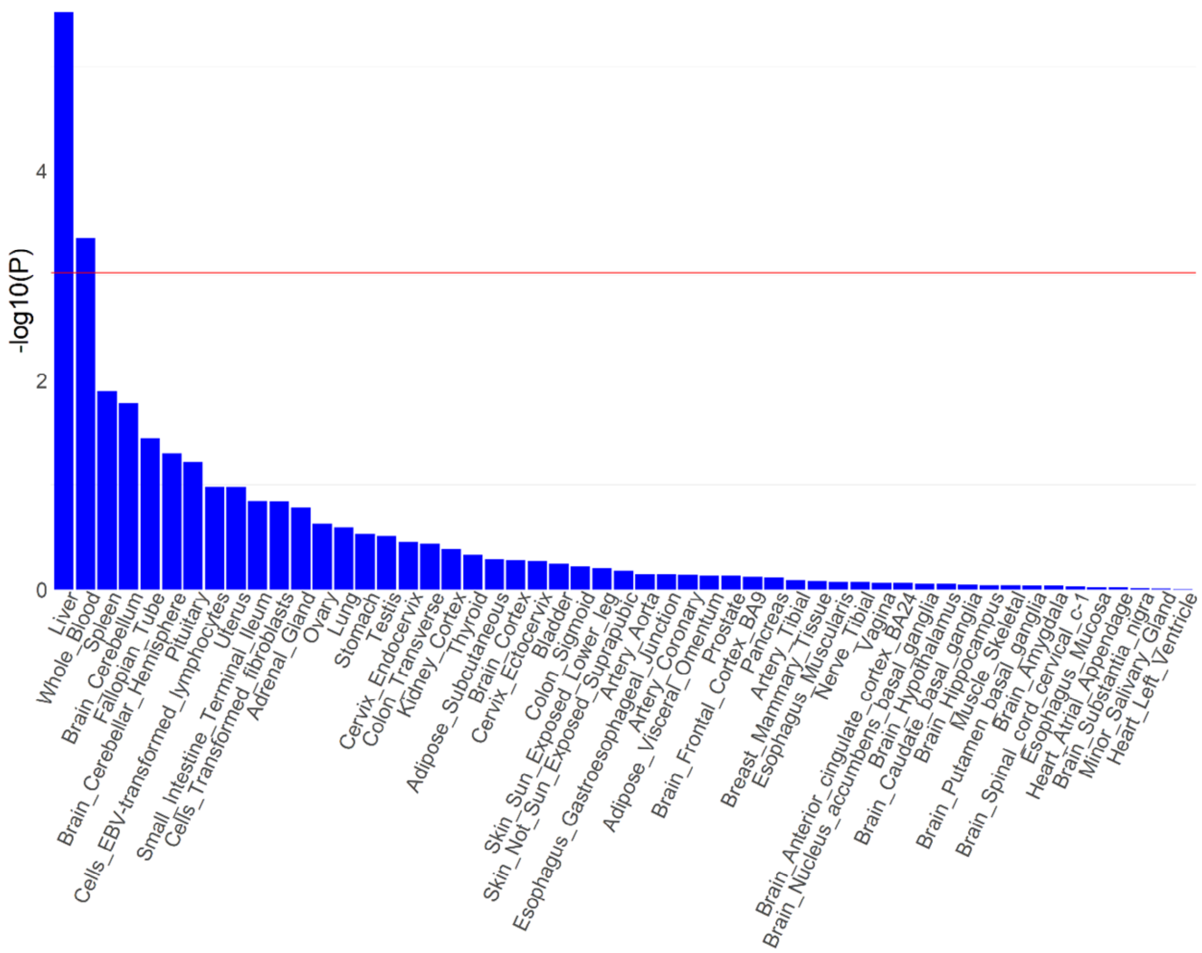


Supplementary Figure 3. MAGMA gene-property analysis results for 53 specific tissue types. The red line indicates the Bonferroni cut off ($p$=9.43 x $10^{-4}$).

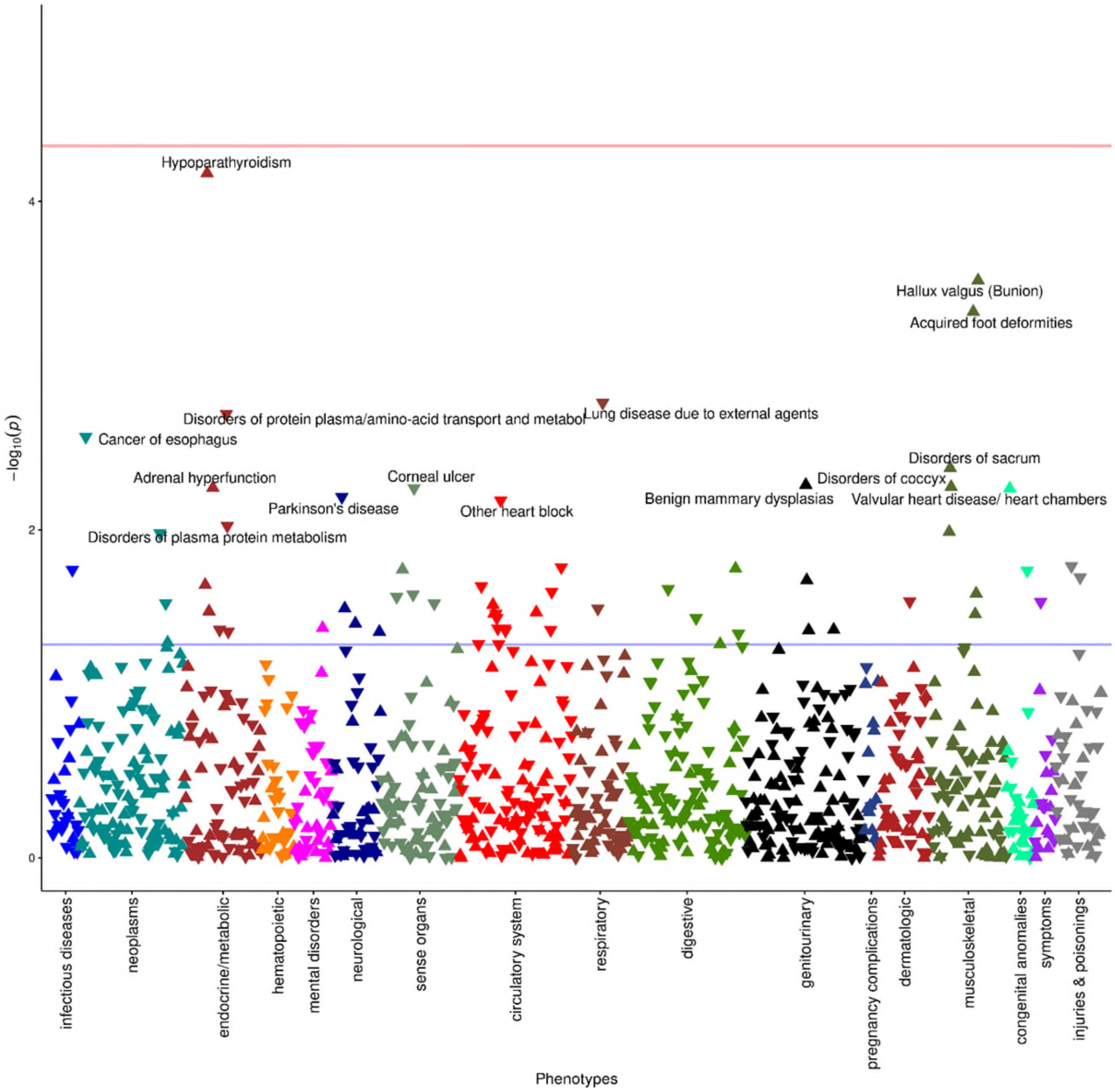


Supplementary Figure 4. CRP gene locus weighted GRS PheWAS Manhattan plot. The red line indicates FDR threshold (q<0.05) and the blue line indicates the nominal threshold (*p*<0.05). The triangle pointing up represents positive association and down a negative association. Phenotypes with *p* <0.01 have been annotated.

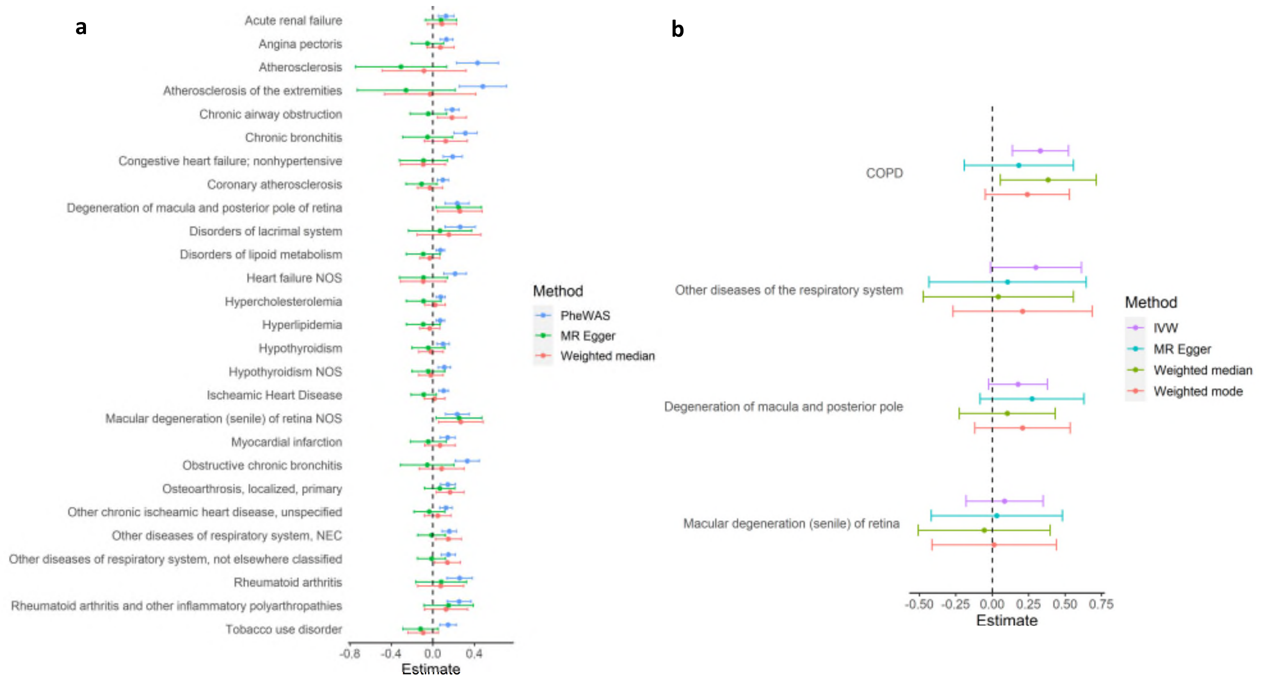


Supplementary Figure 5 a- Forest plot of PheWAS and MR_UKB estimates. PheWAS beta estimates (non-standardised) of 27 disease outcomes are shown with two-sample MR Egger and weighted median estimates. b- Forest plot of replicated MR estimates. Outcomes that were Bonferroni significant for MR_UKB IVW estimate and nominally for weighted median or Egger are presented with MR IVW and sensitivity tests. The points are the beta estimates from the MR analyses and error bars are the 95% confidence intervals.

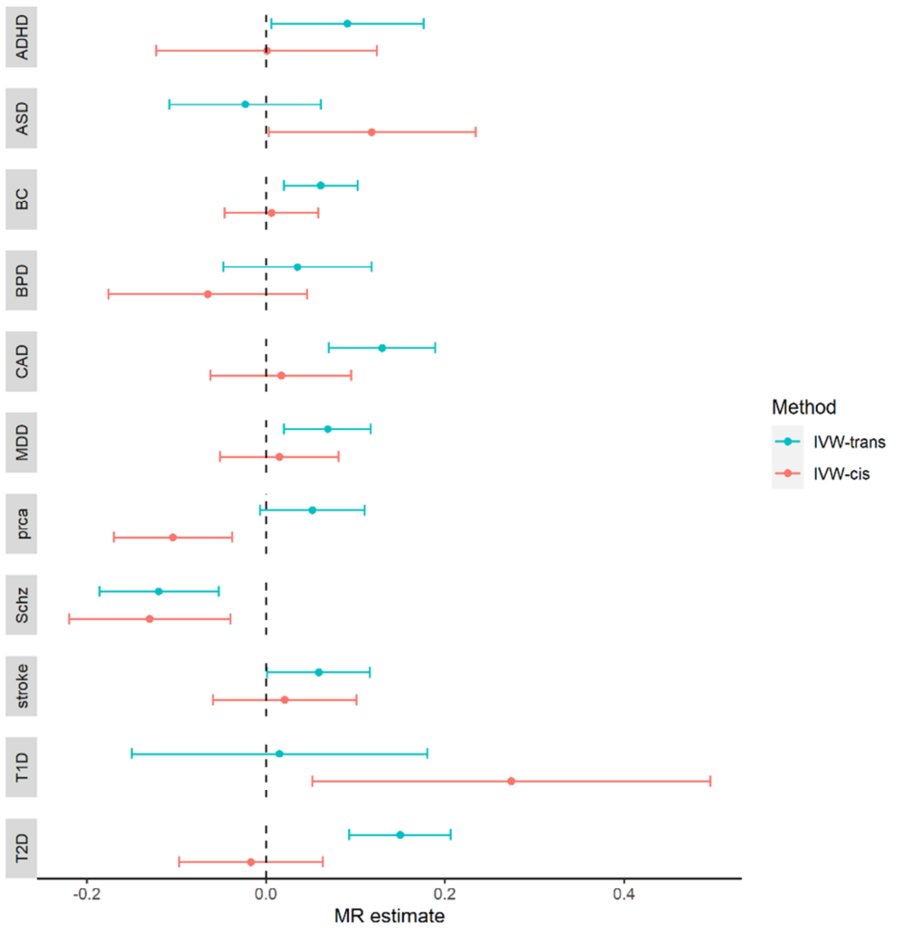


Supplementary Figure 6. Forest plot of cis and trans-acting CRP IVs IVW MR result. The points are the beta estimates from the MR analyses and error bars are the 95% confidence intervals. ADHD = Attention-Deficit/Hyperactivity Disorder, ASD = Autism Spectrum Disorder, BC = Breast Cancer, BPD = Bipolar Disorder, CAD = Coronary Artery Disease, MDD = Major Depressive Disorder, PrC = Prostate Cancer, Schz = Schizophrenia. T1D = Type 1 Diabetes, T2D = Type 2 Diabetes.

## Supplementary Tables

**Supplementary Table 1. UKB study characteristics.**

| Attribute | Value (mean, SD) |
|---|---|
| Age (years) | 56.53 (± 8.10) |
| Female (%) | 54.41% |
| BMI ($kg/m^2$)[1] | 27.39 (± 4.79) |
| CRP (mg/l)[2] | 1.33( IQR 2.1)* |

[1] BMI Body mass index, [2] C reactive protein ,*median and IQR= interquartile range

**Supplementary Table 2. List of immune modulating drugs and immune related diseases and conditions excluded from UKB data.**

| Code | Immunomodulating drug | Use |
|---|---|---|
| 1141172616 | glatiramer | multiple sclerosis |
| 1141172486 | interferon beta | multiple sclerosis |
| 1140923792 | interferon beta- 1b | multiple sclerosis |
| 1141150594 | interferon beta- 1a | multiple sclerosis |
| 1141157420 | interferon beta- 1b product | multiple sclerosis |
| 1140869848 | methotrexate | rheumatoid arthritis /cancers |
| 1141166294 | leflunomide | rheumatoid arthritis / and other autoimmune diseases |
| 1140884308 | hydroxychloroquine | rheumatoid arthritis /malaria/lupus |
| 1140909702 | sulfasalazine | rheumatoid arthritis /chrons disease/ulcerative colitis/ankylosing spondylitis |
| 1140909844 | Ciclosporin | transplant/ rheumatoid arthritis /psoriasis/chrons disease/nephrotic syndrome/ulcerative colitis |
| 1141172620 | Copaxone | multiple sclerosis |
| 1140869930 | azathioprine | chrons/ rheumatoid arthritis /ulcerative cholitis/transplants/ myasthenia gravis/polyarteritis nodosa |
| 1140925978 | mycophenolate | transplant/ myasthenia gravis |

| code | phenotypes to exclude |
|---|---|
| 1234 | adrenocortical insufficiency/Addison’s disease |
| 1261 | multiple sclerosis |
| 1311 | spine arthritis/spondylitis |
| 1313 | ankylosing spondylitis |
| 1322 | myositis/myopathy |
| 1345 | pemphigoid/pemphigus |
| 1372 | vasculitis |
| 1373 | connective tissue disorder |
| 1380 | polyartertis nodosa |
| 1381 | systemic lupus erythematosis |
| 1382 | Sjogrens syndrome/sicca syndrome |
| 1384 | scleroderma/systemic sclerosis |

| | |
|---|---|
| 1437 | myasthenia gravis |
| 1453 | psoriasis |
| 1456 | coeliac disease |
| 1463 | ulcerative colitis |
| 1464 | rheumatoid arthritis |
| 1477 | psoriatic arthropathy |
| 1522 | graves disease |
| 1564 | antiphospholipid syndrome |

**Supplementary Table 3. Information on PheWAS outcomes tested in two-sample MR.**

| Description | Group | N cases | N control | N* | Two-sample MR ID | Trait | Study pmid/author | Population |
|---|---|---|---|---|---|---|---|---|
| Chronic airway obstruction/COPD | respiratory | 2372 | 83986 | 4614 | Finn-a-J10_COPD | Chronic obstructive pulmanary diease,COPD | FinnGen biobank analysis | European |
| Chronic bronchitis | respiratory | 177 | 83986 | 353 | finn-a-J10_BRONCHNAS | Unspecified chronic bronchitis | 2020 FinnGen biobank analysis | European |
| Other diseases of respiratory system, NEC | respiratory | 840 | 95659 | 1665 | finn-a-J10_RESPOTHER | Other diseases of the respiratory system | 2020 FinnGen biobank analysis | European |
| Rheumatoid arthritis | musculoskeletal | 19234 | 61565 | 29311 | ieu-a-833 | Rheumatoid arthritis | 24390342/Okada Y 2014 | Mixed |
| Macular degeneration (senile) of retina NOS | sense organs | 1266 | 47,560 | 2466 | finn-a-CUSTOM_AMD | Wet or dry macular degeneration | FinnGen biobank analysis | European |
| Degeneration of macula and posterior pole of retina | sense organs | 2215 | 90618 | 4324 | finn-a-H7_MACULADEGEN | Degeneration of macula and posterior pole | FinnGen biobank analysis | European |
| Ischemic Heart Disease | circulatory system | 63746 | 130681 | 85692 | ieu-a-7 | Coronary heart disease (Ischeamic heart disease) | 26343387/Nikpay 2015 | Mixed |
| Other chronic ischeamic heart disease, unspecified | circulatory system | 21362 | 75137 | 33266 | finn-a-I9_OTHHEART | Other heart disease (Ischeamic heart disease) | FinnGen biobank analysis | European |
| Angina pectoris | circulatory system | 6382 | 85760 | 11880 | finn-a-I9_ANGINA | Angina pectoris | FinnGen biobank analysis | European |
| Atherosclerosis | circulatory system | 2196 | 92349 | 4290 | finn-a-I9_ATHSCLE | Atherosclerosis, excluding cerebral, coronary and PAD | FinnGen biobank analysis | European |

| | | | | | | | | |
|---|---|---|---|---|---|---|---|---|
| Atherosclerosis of the extremities | circulatory system | 2181 | 75420 | 4239 | finn-a-DM_PERIPHATHERO | Peripheral atherosclerosis | FinnGen biobank analysis | European |
| Coronary atherosclerosis | circulatory system | 7661 | 85760 | 14066 | finn-a-I9_CORATHER | Coronary atherosclerosis | FinnGen biobank analysis | European |
| Heart failure NOS | circulatory system | 8016 | 75137 | 14487 | finn-a-HEARTFAIL | Heart failure | FinnGen biobank analysis | European |
| Congestive heart failure; non-hypertensive | circulatory system | 9413 | 203040 | 17992 | bbj-a-109 | Congestive heart failure | Ishigaki K 2019 (east Asian sample) | East Asian |
| Myocardial infarction | circulatory system | 43676 | 128199 | 65155 | ieu-a-798 | Myocardial infarction | Nikpay 2015/26343387 | Mixed |
| Hypothyroidism | endocrine/metabolic | 10211 | 32736 | 15567 | finn-a-HYPOTHYROIDISM | Hypothyroidism (congenital or acquired) | FinnGen biobank analysis | European |
| Hypothyroidism NOS | endocrine/metabolic | 10192 | 84518 | 18190 | finn-a-E4_HYTHYNAS | Hypothyroidism,other/unspecified | FinnGen biobank analysis | European |
| Disorders of lacrimal system | sense organs | 2256 | 90757 | 4403 | finn-a-H7_LACRIMALSYSTEM | Disorders of lacrimal system | FinnGen biobank analysis | European |
| Hypercholesterolemia | endocrine/metabolic | 3262 | 91366 | 6299 | finn-a-E4_HYPERCHOL | Pure hypercholesterolaemia | FinnGen biobank analysis | European |
| Disorders of lipoid metabolism | endocrine/metabolic | 5133 | 88782 | 9705 | finn-a-E4_LIPOPROT | Disorders of lipoprotein metabolism and other lipidaemias | FinnGen biobank analysis | European |
| Hyperlipidemia | endocrine/metabolic | 1539 | 91366 | 3027 | finn-a-E4_HYPERLIPNAS | Hyperlipidaemia, other/unspecified | FinnGen biobank analysis | European |

| | | | | | | | | |
|---|---|---|---|---|---|---|---|---|
| Acute renal failure | genitourinary | 584 | 94913 | 1161 | finn-a-N14_ACUTERENFAIL | Acute renal failure | FinnGen biobank analysis | European |

*For binary traits, N is the effective sample size (N_eff = 2/(1/cases + 1/controls) (see e.g. https://doi.org/10.1038/nprot.2014.071).

**Supplementary Table 4. Information on outcomes tested in MR.**

| Outcome | Abbreviation | N case | N control | N* | Source | doi |
|---|---|---|---|---|---|---|
| Breast cancer | | 108067 | 88386 | 97241 | Michailidou et al. 2017 | 10.1038/nature24284 |
| Prostate cancer | | 79194 | 61112 | 68988 | Schumacher et al. 2018 | 10.1038/s41588-018-0142-8 |
| Attention-deficit/hyperactivity disorder | ADHD | 20183 | 35191 | 25653 | Demontis et al. 2019 | 10.1038/s41588-018-0269-7 |
| Autism spectrum disorder | ASD | 18381 | 27969 | 22183 | Grove et al. 2019 | 10.1038/s41588-019-0344-8 |
| Bipolar disorder | BPD | 20352 | 31358 | 24684 | Stahl et al. 2019 | 10.1038/s41588-019-0397-8 |
| Major depressive disorder | MDD | 135458 | 344901 | 194520 | Wray et al. 2018 | 10.1038/s41588-018-0090-3 |
| Schizophrenia | Schz | 36989 | 113075 | 55743 | Ripke et al. 2014 | 10.1038/nature13595 |
| Type 2 diabetes | T2D | 62892 | 596424 | 113786 | Xue et al. 2018 | 10.1038/s41467-018-04951-w |
| Coronary artery disease | CAD | 60801 | 123504 | 81486 | Nikpay et al. 2015 | 10.1038/ng.3396 |
| Stroke | | 40585 | 406111 | 73795 | Malik et al. 2015 | 10.1038/s41588-018-0058-3 |
| Type I diabetes | T1D | 5913 | 8828 | 7082 | Censin et al. 2017 | 10.1371/journal.pmed.1002362 |

*For binary traits, N is the effective sample size (N_eff = 2/(1/cases + 1/controls) (see e.g. https://doi.org/10.1038/nprot.2014.071).

**Supplementary Table 5. Previously identified sentinel variants associated to CRP in the current UKB GWAS loci.**

| SNP | Chr | BP | Effect allele | Ref. allele | Beta | SE | P value | Closest Gene |
|---|---|---|---|---|---|---|---|---|
| rs4129267 | 1 | 154426264 | C | T | 0.094 | 0.002 | < 4.94E-324 | *IL6R* |
| rs2794520 | 1 | 159678816 | C | T | 0.173 | 0.002 | < 4.94E-324 | *CRP* |
| rs10925027 | 1 | 247612562 | T | C | 0.033 | 0.002 | 5.90E-56 | *NLRP3* |
| rs75460349 | 1 | 27180088 | A | C | 0.097 | 0.007 | 4.50E-44 | *ZDHHC18* |
| rs2293476 | 1 | 40036847 | G | C | -0.029 | 0.002 | 1.70E-32 | *PABPC4* |
| rs1805096 | 1 | 66102257 | G | A | 0.121 | 0.002 | < 4.94E-324 | *LEPR* |
| rs469772 | 1 | 91530305 | C | T | 0.035 | 0.003 | 6.90E-41 | *ZNF644* |
| rs9284725 | 2 | 102744854 | C | A | 0.020 | 0.002 | 4.10E-16 | *IL1R1* |
| rs13409371 | 2 | 113838145 | G | A | -0.044 | 0.002 | 1.40E-95 | *IL1F10* |
| rs1441169 | 2 | 214033530 | A | G | 0.021 | 0.002 | 3.60E-23 | *IKZF2* |
| rs1260326 | 2 | 27730940 | T | C | 0.078 | 0.002 | 8.20E-291 | *GCKR* |
| rs12995480 | 2 | 629881 | T | C | -0.024 | 0.003 | 1.80E-18 | *TMEM18* |
| rs4246598 | 2 | 88438050 | C | A | -0.013 | 0.002 | 1.40E-09 | *FABP1* |
| rs687339 | 3 | 135932359 | C | T | 0.026 | 0.003 | 3.90E-24 | *MSL2* |
| rs1514895 | 3 | 170705693 | A | G | -0.017 | 0.002 | 3.70E-14 | *EIF5A2* |
| 3:47431869_GTCT_G | 3 | 47431869 | GTCT | G | -0.010 | 0.002 | 1.80E-06 | *PTPN23* |
| rs2352975 | 3 | 49891885 | T | C | -0.021 | 0.002 | 4.50E-22 | *TRAIP* |
| rs17658229 | 5 | 172191052 | T | C | -0.038 | 0.005 | 2.30E-14 | *DUSP1* |
| rs12202641 | 6 | 116314634 | C | T | 0.017 | 0.002 | 9.00E-17 | *FRK* |
| rs1490384 | 6 | 126851160 | C | T | 0.029 | 0.002 | 4.50E-42 | *C6orf173* |
| rs9385532 | 6 | 130371227 | T | C | -0.023 | 0.002 | 4.50E-24 | *L3MBTL3* |
| rs9271608* | 6 | 32591588 | A | G | -0.039 | 0.003 | 4.90E-48 | *HLA-DQA1* |
| rs1880241 | 7 | 22759469 | A | G | 0.024 | 0.002 | 1.50E-29 | *IL6* |
| rs2710804 | 7 | 36084529 | T | C | -0.025 | 0.002 | 3.30E-30 | *KIAA1706* |
| rs13233571 | 7 | 72971231 | C | T | 0.033 | 0.003 | 2.40E-24 | *BCL7B* |
| rs7795281 | 7 | 74122854 | A | G | 0.013 | 0.002 | 5.90E-08 | *GTF2I* |
| rs1736060 | 8 | 11664738 | C | T | -0.023 | 0.002 | 7.40E-26 | *FDFT1* |
| rs2064009 | 8 | 117007850 | C | T | -0.026 | 0.002 | 3.20E-35 | *TRPS1* |
| rs2891677 | 8 | 126344208 | C | T | -0.022 | 0.002 | 1.90E-24 | *NSMCE2* |

| | | | | | | | | |
|---|---|---|---|---|---|---|---|---|
| rs4841132 | 8 | 9183596 | A | G | -0.083 | 0.004 | 3.00E-113 | *PPP1R3B* |
| rs643434 | 9 | 136142355 | G | A | -0.022 | 0.002 | 1.60E-23 | *ABO* |
| rs1051338 | 10 | 91007360 | T | G | -0.028 | 0.002 | 3.60E-33 | *LIPA* |
| rs10832027 | 11 | 13357183 | G | A | -0.030 | 0.002 | 2.50E-39 | *ARNTL* |
| rs10838687 | 11 | 47312892 | T | G | 0.029 | 0.003 | 2.70E-27 | *MADD* |
| rs1582763 | 11 | 60021948 | G | A | 0.020 | 0.002 | 3.80E-21 | *MS4A4A* |
| rs7121935 | 11 | 72496148 | G | A | 0.015 | 0.002 | 1.10E-11 | *STARD10* |
| rs10778215 | 12 | 103537266 | T | A | 0.018 | 0.002 | 2.90E-18 | *ASCL1* |
| rs7310409 | 12 | 121424861 | A | G | -0.150 | 0.002 | < 4.94E-324 | *HNF1A* |
| rs11108056 | 12 | 95855385 | C | G | 0.026 | 0.002 | 1.70E-35 | *METAP2* |
| rs2239222 | 14 | 73011885 | A | G | -0.035 | 0.002 | 2.50E-57 | *RGS6* |
| rs112635299 | 14 | 94838142 | G | T | 0.096 | 0.007 | 2.00E-40 | *SERPINA1/2* |
| rs4774590 | 15 | 51745277 | G | A | 0.006 | 0.002 | 0.0056 | *DMXL2* |
| rs1189402 | 15 | 53728154 | A | G | 0.015 | 0.002 | 2.00E-12 | *ONECUT1* |
| rs340005 | 15 | 60878030 | G | A | -0.033 | 0.002 | 4.70E-52 | *RORA* |
| rs10521222 | 16 | 51158710 | C | T | 0.107 | 0.005 | 4.90E-106 | *SALL1* |
| rs1558902 | 16 | 53803574 | T | A | -0.025 | 0.002 | 2.80E-31 | *FTO* |
| rs178810 | 17 | 16097430 | C | T | -0.015 | 0.002 | 2.70E-13 | *NCOR1* |
| 17:58001690_GA_G | 17 | 58001690 | GA | G | 0.018 | 0.002 | 1.60E-17 | *RPS6KB1* |
| rs10512597 | 17 | 72699833 | T | C | -0.030 | 0.003 | 3.90E-29 | *CD300LF,RAB37* |
| rs2852151 | 18 | 12841176 | G | A | -0.019 | 0.002 | 8.70E-18 | *PTPN2* |
| rs4092465 | 18 | 55080437 | A | G | -0.027 | 0.002 | 1.40E-34 | *ONECUT2* |
| rs12960928 | 18 | 57897803 | T | C | -0.018 | 0.002 | 6.30E-14 | *MC4R* |
| rs4420638 | 19 | 45422946 | A | G | 0.221 | 0.003 | < 4.94E-324 | *APOC1* |
| rs1800961 | 20 | 43042364 | C | T | 0.110 | 0.006 | 2.00E-72 | *HNF4A* |
| rs2315008 | 20 | 62343956 | T | G | -0.020 | 0.002 | 2.50E-18 | *ZGPAT* |
| rs2836878 | 21 | 40465534 | G | A | 0.034 | 0.002 | 3.50E-46 | *DSCR2* |
| rs6001193 | 22 | 39074737 | A | G | 0.030 | 0.002 | 4.70E-43 | *TOMM22* |
| rs9611441 | 22 | 41339367 | G | C | 0.014 | 0.002 | 1.50E-11 | *XPNPEP3* |

* This variant is replicated in the UKB GWAS but HLA region was excluded in loci definition so was not found in UKB GWAS loci.

Variants listed were genome-wide significant variants replicated in current UKB GWAS at p <0.01 with concordant effect direction.

**Supplementary Table 6. MAGMA gene set analysis Bonferroni significant results for curated gene sets.**

| Gene sets* | N Genes | Beta | Beta STD | SE | P value |
|---|---|---|---|---|---|
| **GO_bp:go_positive_regulation_of_gene_expression** | 1825 | 0.283 | 0.083 | 0.036 | 2.55E-15 |
| GO_bp:go_positive_regulation_of_biosynthetic_process | 1844 | 0.274 | 0.081 | 0.036 | 1.09E-14 |
| GO_bp:go_positive_regulation_of_rna_biosynthetic_process | 1505 | 0.299 | 0.080 | 0.039 | 1.53E-14 |
| **GO_bp:go_positive_regulation_of_transcription_by_rna_polymerase_ii** | 1123 | 0.339 | 0.080 | 0.046 | 4.71E-14 |
| GO_bp:go_regulation_of_lipid_localization | 134 | 0.876 | 0.073 | 0.137 | 8.87E-11 |
| GO_bp:go_negative_regulation_of_biosynthetic_process | 1420 | 0.244 | 0.064 | 0.040 | 8.19E-10 |
| GO_mf:go_regulatory_region_nucleic_acid_binding | 890 | 0.308 | 0.065 | 0.051 | 8.84E-10 |
| **Curated_gene_sets:roversi_glioma_copy_number_up** | 97 | 1.123 | 0.080 | 0.188 | 1.15E-09 |
| GO_mf:go_dna_binding_transcription_factor_activity | 1603 | 0.238 | 0.066 | 0.041 | 2.23E-09 |
| Curated_gene_sets:reactome_interleukin_36_pathway | 7 | 4.275 | 0.082 | 0.742 | 4.21E-09 |
| GO_bp:go_regulation_of_interleukin_1_mediated_signaling_pathway | 7 | 3.588 | 0.069 | 0.647 | 1.46E-08 |
| GO_bp:go_negative_regulation_of_interleukin_1_mediated_signaling_pathway | 5 | 4.412 | 0.071 | 0.811 | 2.66E-08 |
| GO_mf:go_sequence_specific_dna_binding | 1060 | 0.254 | 0.058 | 0.047 | 3.30E-08 |
| GO_bp:go_negative_regulation_of_rna_biosynthetic_process | 1138 | 0.240 | 0.057 | 0.045 | 4.77E-08 |
| GO_mf:go_sequence_specific_double_stranded_dna_binding | 821 | 0.275 | 0.056 | 0.053 | 1.04E-07 |
| GO_bp:go_t_cell_differentiation_involved_in_immune_response | 63 | 1.015 | 0.058 | 0.196 | 1.15E-07 |
| GO_bp:go_circadian_regulation_of_gene_expression | 54 | 1.020 | 0.054 | 0.198 | 1.28E-07 |
| GO_bp:go_negative_regulation_of_receptor_signaling_pathway_via_stat | 20 | 1.721 | 0.056 | 0.335 | 1.36E-07 |
| GO_bp:go_negative_regulation_of_transcription_by_rna_polymerase_ii | 771 | 0.273 | 0.054 | 0.053 | 1.68E-07 |
| GO_mf:go_double_stranded_dna_binding | 900 | 0.256 | 0.054 | 0.050 | 1.78E-07 |
| GO_bp:go_inflammatory_response | 658 | 0.297 | 0.054 | 0.060 | 3.63E-07 |
| GO_bp:go_t_cell_activation_involved_in_immune_response | 96 | 0.837 | 0.059 | 0.169 | 3.67E-07 |
| GO_bp:go_positive_regulation_of_lipid_localization | 80 | 0.838 | 0.054 | 0.170 | 3.95E-07 |
| GO_bp:go_regulation_of_lipid_transport | 105 | 0.787 | 0.058 | 0.160 | 4.18E-07 |
| GO_bp:go_regulation_of_glucocorticoid_receptor_signaling_pathway | 7 | 3.113 | 0.060 | 0.636 | 4.97E-07 |
| GO_bp:go_t_helper_17_type_immune_response | 26 | 1.434 | 0.053 | 0.297 | 7.08E-07 |
| GO_mf:go_proximal_promoter_sequence_specific_dna_binding | 515 | 0.324 | 0.052 | 0.068 | 8.20E-07 |
| GO_bp:go_negative_regulation_of_relaxation_of_muscle | 5 | 4.148 | 0.067 | 0.867 | 8.53E-07 |
| GO_mf:go_interleukin_1_receptor_binding | 16 | 1.977 | 0.057 | 0.416 | 1.02E-06 |

| | | | | | |
|---|---|---|---|---|---|
| GO_mf:go_complement_component_c1q_binding | 7 | 3.043 | 0.058 | 0.641 | 1.05E-06 |
| GO_bp:go_positive_regulation_of_t_cell_mediated_immunity | 37 | 1.253 | 0.055 | 0.266 | 1.26E-06 |
| GO_bp:go_positive_regulation_of_t_cell_mediated_immunity | 1698 | 0.173 | 0.049 | 0.037 | 1.41E-06 |
| Curated_gene_sets:reactome_signaling_by_leptin | 11 | 2.112 | 0.051 | 0.456 | 1.78E-06 |
| GO_bp:go_response_to_lipid | 879 | 0.230 | 0.048 | 0.050 | 1.86E-06 |
| GO_bp:go_interleukin_6_mediated_signaling_pathway | 19 | 1.446 | 0.046 | 0.313 | 1.98E-06 |
| GO_bp:go_positive_regulation_of_lipid_transport | 60 | 0.935 | 0.052 | 0.203 | 2.07E-06 |
| Curated_gene_sets:pid_il12_2pathway | 59 | 0.906 | 0.050 | 0.198 | 2.26E-06 |
| GO_bp:go_response_to_molecule_of_bacterial_origin | 326 | 0.372 | 0.048 | 0.081 | 2.28E-06 |
| GO_bp:go_lymphocyte_activation_involved_in_immune_response | 166 | 0.572 | 0.053 | 0.125 | 2.42E-06 |
| Curated_gene_sets:dacosta_uv_response_via_ercc3_common_dn | 453 | 0.344 | 0.052 | 0.076 | 2.89E-06 |
| GO_bp:go_regulation_of_lipid_storage | 45 | 1.022 | 0.050 | 0.226 | 2.95E-06 |
| Curated_gene_sets:biocarta_circadian_pathway | 6 | 3.097 | 0.055 | 0.684 | 3.00E-06 |

GO, Gene ontology; bp, biological processes; mf, molecular functions.

* The gene sets in bold represent the previously reported MAGMA gene sets from prior CRP GWAS

Bonferroni significance at p < 3.23 x 10-6

**Supplementary Table 7. MAGMA Gene-set association for tissue specific gene expression results, 53 tissue.**

| Tissue | Beta | SE | P value |
|---|---|---|---|
| **Liver** | **0.050** | **0.011** | **3.04E-06** |
| **Whole_Blood** | **0.033** | **0.010** | **4.42E-04** |
| Spleen | 0.028 | 0.012 | 0.013 |
| Brain_Cerebellum | 0.024 | 0.011 | 0.017 |
| Fallopian_Tube | 0.036 | 0.020 | 0.036 |
| Brain_Cerebellar_Hemisphere | 0.018 | 0.011 | 0.050 |
| Pituitary | 0.024 | 0.016 | 0.061 |
| Cells_EBV-transformed_lymphocytes | 0.011 | 0.009 | 0.104 |
| Uterus | 0.024 | 0.019 | 0.105 |
| Small_Intestine_Terminal_Ileum | 0.017 | 0.016 | 0.144 |
| Cells_Transformed_fibroblasts | 0.012 | 0.012 | 0.145 |
| Adrenal_Gland | 0.017 | 0.017 | 0.165 |
| Ovary | 0.012 | 0.016 | 0.236 |
| Lung | 0.010 | 0.016 | 0.257 |
| Stomach | 0.011 | 0.020 | 0.298 |
| Testis | 0.005 | 0.010 | 0.309 |
| Cervix_Endocervix | 0.008 | 0.020 | 0.350 |
| Colon_Transverse | 0.007 | 0.019 | 0.365 |
| Kidney_Cortex | 0.004 | 0.016 | 0.408 |
| Thyroid | 0.001 | 0.017 | 0.468 |
| Adipose_Subcutaneous | -0.001 | 0.019 | 0.516 |
| Brain_Cortex | -0.001 | 0.012 | 0.530 |
| Cervix_Ectocervix | -0.002 | 0.022 | 0.538 |
| Bladder | -0.003 | 0.020 | 0.564 |
| Colon_Sigmoid | -0.005 | 0.021 | 0.596 |
| Skin_Sun_Exposed_Lower_leg | -0.004 | 0.014 | 0.627 |
| Skin_Not_Sun_Exposed_Suprapubic | -0.006 | 0.014 | 0.659 |
| Artery_Aorta | -0.010 | 0.018 | 0.715 |
| Esophagus_Gastroesophageal_Junction | -0.013 | 0.022 | 0.716 |
| Artery_Coronary | -0.012 | 0.020 | 0.729 |
| Adipose_Visceral_Omentum | -0.012 | 0.019 | 0.737 |
| Prostate | -0.013 | 0.021 | 0.739 |
| Brain_Frontal_Cortex_BA9 | -0.008 | 0.012 | 0.762 |
| Pancreas | -0.011 | 0.015 | 0.774 |
| Artery_Tibial | -0.016 | 0.018 | 0.823 |
| Breast_Mammary_Tissue | -0.021 | 0.022 | 0.835 |
| Esophagus_Muscularis | -0.022 | 0.021 | 0.849 |
| Nerve_Tibial | -0.018 | 0.018 | 0.852 |
| Vagina | -0.021 | 0.019 | 0.861 |
| Brain_Anterior_cingulate_cortex_BA24 | -0.014 | 0.013 | 0.870 |
| Brain_Nucleus_accumbens_basal_ganglia | -0.016 | 0.013 | 0.888 |
| Brain_Hypothalamus | -0.017 | 0.014 | 0.888 |
| Brain_Caudate_basal_ganglia | -0.018 | 0.014 | 0.904 |

| | | | |
|---|---|---|---|
| Brain_Hippocampus | -0.018 | 0.014 | 0.907 |
| Muscle_Skeletal | -0.016 | 0.012 | 0.912 |
| Brain_Putamen_basal_ganglia | -0.020 | 0.014 | 0.924 |
| Brain_Amygdala | -0.019 | 0.014 | 0.924 |
| Brain_Spinal_cord_cervical_c-1 | -0.023 | 0.015 | 0.935 |
| Esophagus_Mucosa | -0.021 | 0.013 | 0.946 |
| Heart_Atrial_Appendage | -0.027 | 0.017 | 0.947 |
| Brain_Substantia_nigra | -0.027 | 0.015 | 0.965 |
| Minor_Salivary_Gland | -0.035 | 0.017 | 0.981 |
| Heart_Left_Ventricle | -0.038 | 0.016 | 0.992 |

Bonferroni significant tissue in bold with p < 9.43 x 10-4

**Supplementary Table 8. Significant enriched tissue from DEPICT analysis.**

| MeSH term* | Name | MeSH first level term | MeSH second level term | P value | False discovery rate |
|---|---|---|---|---|---|
| **A11.118.637.555.567.562.440** | **Precursor Cells B Lymphoid** | **Cells** | **Blood Cells** | **8.21E-07** | <0.01 |
| **A11.872.378.294** | **Lymphoid Progenitor Cells** | **Cells** | **Stem Cells** | **8.21E-07** | <0.01 |
| **A02.835.583.443.800.800** | **Synovial Fluid** | **Musculoskeletal System** | **Skeleton** | **1.46E-05** | <0.01 |
| **A03.620** | **Liver** | **Digestive System** | **Liver** | **2.11E-05** | <0.01 |
| **A15.145** | **Blood** | **Hemic and Immune Systems** | **Blood** | **2.66E-05** | <0.01 |
| **A15.145.229** | **Blood Cells** | **Hemic and Immune Systems** | **Blood** | **6.51E-05** | <0.01 |
| **A15.378.316** | **Bone Marrow Cells** | **Hemic and Immune Systems** | **Hematopoietic System** | **0.000122** | <0.01 |
| **A15.378** | **Hematopoietic System** | **Hemic and Immune Systems** | **Hematopoietic System** | **0.000122** | <0.01 |
| A15.382.490.315.583 | Neutrophils | Hemic and Immune Systems | Immune System | 0.000401 | <0.01 |
| A11.118.637.415 | Granulocytes | Cells | Blood Cells | 0.000433 | <0.01 |
| A15.382.680 | Phagocytes | Hemic and Immune Systems | Immune System | 0.000549 | <0.01 |
| A11.627 | Myeloid Cells | Cells | Myeloid Cells | 0.000642 | <0.01 |
| A11.066 | Antigen Presenting Cells | Cells | Antigen-Presenting Cells | 0.000789 | <0.01 |
| A15.382.812.260 | Dendritic Cells | Hemic and Immune Systems | Immune System | 0.000789 | <0.01 |
| A06.407.071.140 | Adrenal Cortex | Endocrine System | Endocrine Glands | 0.000815 | <0.01 |
| A02.835.232.043.300 | Foot Bones | Musculoskeletal System | Skeleton | 0.00104 | <0.01 |
| A02.835.232.043.300.710 | Tarsal Bones | Musculoskeletal System | Skeleton | 0.00104 | <0.01 |
| A02.835.232.043 | Bones of Lower Extremity | Musculoskeletal System | Skeleton | 0.00108 | <0.01 |
| A11.118.637 | Leukocytes | Cells | Blood Cells | 0.00112 | <0.01 |
| A15.382.520.604.800 | Palatine Tonsil | Hemic and Immune Systems | Immune System | 0.00113 | <0.01 |
| A04.623.603 | Oropharynx | Respiratory System | Pharynx | 0.00113 | <0.01 |
| A05.810.890 | Urinary Bladder | Urogenital System | Urinary Tract | 0.00117 | <0.01 |
| A15.145.300 | Fetal Blood | Hemic and Immune Systems | Blood | 0.0014 | <0.01 |
| A15.378.316.580 | Monocytes | Hemic and Immune Systems | Hematopoietic System | 0.00142 | <0.01 |
| A06.407.071 | Adrenal Glands | Endocrine System | Endocrine Glands | 0.00188 | <0.01 |
| A09.371.337 | Eyelids | Sense Organs | Eye | 0.00193 | <0.01 |
| A09.371.337.168 | Conjunctiva | Sense Organs | Eye | 0.00193 | <0.01 |
| A09.371.060 | Anterior Eye Segment | Sense Organs | Eye | 0.00221 | <0.01 |

| | | | | | |
|---|---|---|---|---|---|
| A15.382.812 | Mononuclear Phagocyte System | Hemic and Immune Systems | Immune System | 0.0024 | <0.01 |
| A05.360.319.114.373 | Fallopian Tubes | Urogenital System | Genitalia | 0.00432 | <0.05 |
| A15.382.520.604.700 | Spleen | Hemic and Immune Systems | Immune System | 0.00538 | <0.05 |
| A10.272 | Epithelium | Tissues | Epithelium | 0.00545 | <0.05 |
| A15.145.229.637.555 | Leukocytes Mononuclear | Hemic and Immune Systems | Blood | 0.00578 | <0.05 |
| A15.382.490.555.567.537 | Killer Cells Natural | Hemic and Immune Systems | Immune System | 0.00614 | <0.05 |
| A09.531 | Nose | Sense Organs | Nose | 0.00683 | <0.05 |
| A10.615.550.760 | Respiratory Mucosa | Tissues | Membranes | 0.00683 | <0.05 |
| A04.531.520 | Nasal Mucosa | Respiratory System | Nose | 0.00683 | <0.05 |
| A11.118.637.555.567.569 | T Lymphocytes | Cells | Blood Cells | 0.00831 | <0.05 |
| A02.165 | Cartilage | Musculoskeletal System | Cartilage | 0.00969 | <0.05 |
| A11.436 | Epithelial Cells | Cells | Epithelial Cells | 0.01 | <0.05 |

*Medical Subject Heading (MeSH)

Significance determined by FDR q<0.05

**Supplementary Table 9. Disease outcomes associated with weighted GRS of CRP in PheWAS analysis.**

| Phecode | Outcome description | Group | OR | Beta | SE | Lower | Upper | P value | N total | N cases | N controls | Bonferroni | False discover rate |
|---|---|---|---|---|---|---|---|---|---|---|---|---|---|
| 496 | Chronic airway obstruction | respiratory | 1.046 | 0.045 | 0.008 | 1.030 | 1.062 | 1.19E-08 | 327982 | 17567 | 310415 | TRUE | TRUE |
| 496.21 | Obstructive chronic bronchitis | respiratory | 1.083 | 0.080 | 0.014 | 1.054 | 1.114 | 1.71E-08 | 315551 | 5136 | 310415 | TRUE | TRUE |
| 496.2 | Chronic bronchitis | respiratory | 1.078 | 0.075 | 0.014 | 1.050 | 1.108 | 3.79E-08 | 315919 | 5504 | 310415 | TRUE | TRUE |
| 411 | Ischemic Heart Disease | circulatory system | 1.025 | 0.025 | 0.006 | 1.014 | 1.036 | 7.92E-06 | 327162 | 40105 | 287057 | TRUE | TRUE |
| 519.8 | Other diseases of respiratory system, NEC | respiratory | 1.039 | 0.038 | 0.009 | 1.021 | 1.056 | 9.47E-06 | 328108 | 14515 | 313593 | TRUE | TRUE |
| 714 | Rheumatoid arthritis and other inflammatory polyarthropathies | musculoskeletal | 1.063 | 0.061 | 0.014 | 1.033 | 1.093 | 1.89E-05 | 322471 | 5112 | 317359 | TRUE | TRUE |
| 411.3 | Angina pectoris | circulatory system | 1.032 | 0.032 | 0.007 | 1.017 | 1.047 | 2.16E-05 | 307501 | 20444 | 287057 | TRUE | TRUE |
| 519 | Other diseases of respiratory system, not elsewhere classified | respiratory | 1.036 | 0.036 | 0.008 | 1.019 | 1.054 | 2.21E-05 | 328573 | 14980 | 313593 | TRUE | TRUE |
| 440 | Atherosclerosis | circulatory system | 1.109 | 0.103 | 0.025 | 1.056 | 1.164 | 3.02E-05 | 317387 | 1660 | 315727 | TRUE | TRUE |
| 440.2 | Atherosclerosis of the extremities | circulatory system | 1.123 | 0.116 | 0.028 | 1.063 | 1.186 | 3.36E-05 | 317029 | 1302 | 315727 | TRUE | TRUE |
| 714.1 | Rheumatoid arthritis | musculoskeletal | 1.064 | 0.062 | 0.015 | 1.033 | 1.096 | 3.93E-05 | 321884 | 4525 | 317359 | TRUE | TRUE |
| 411.8 | Other chronic ischemic heart disease, unspecified | circulatory system | 1.031 | 0.030 | 0.007 | 1.016 | 1.046 | 4.11E-05 | 308289 | 21232 | 287057 | TRUE | TRUE |
| 362.29 | Macular degeneration (senile) of retina NOS | sense organs | 1.058 | 0.057 | 0.014 | 1.030 | 1.088 | 5.15E-05 | 317190 | 5314 | 311876 | FALSE | TRUE |
| 428 | Congestive heart failure; nonhypertensive | circulatory system | 1.047 | 0.046 | 0.011 | 1.024 | 1.070 | 5.47E-05 | 327840 | 8256 | 319584 | FALSE | TRUE |

| | | | | | | | | | | | | | |
|---|---|---|---|---|---|---|---|---|---|---|---|---|---|
| 362.2 | Degeneration of macula and posterior pole of retina | sense organs | 1.058 | 0.056 | 0.014 | 1.029 | 1.087 | 5.64E-05 | 317193 | 5317 | 311876 | FALSE | TRUE |
| 740.11 | Osteoarthrosis, localized, primary | musculoskeletal | 1.035 | 0.035 | 0.009 | 1.018 | 1.053 | 6.38E-05 | 283671 | 14428 | 269243 | FALSE | TRUE |
| 411.2 | Myocardial infarction | circulatory system | 1.035 | 0.034 | 0.009 | 1.017 | 1.053 | 1.08E-04 | 301218 | 14161 | 287057 | FALSE | TRUE |
| 428.2 | Heart failure NOS | circulatory system | 1.053 | 0.051 | 0.013 | 1.026 | 1.081 | 1.16E-04 | 325426 | 5842 | 319584 | FALSE | TRUE |
| 318 | Tobacco use disorder | mental disorders | 1.036 | 0.035 | 0.010 | 1.016 | 1.056 | 3.41E-04 | 322768 | 10831 | 311937 | FALSE | TRUE |
| 244.4 | Hypothyroidism NOS | endocrine/ metabolic | 1.027 | 0.026 | 0.007 | 1.012 | 1.042 | 4.06E-04 | 324451 | 20075 | 304376 | FALSE | TRUE |
| 375 | Disorders of lacrimal system | sense organs | 1.065 | 0.063 | 0.018 | 1.028 | 1.103 | 4.28E-04 | 323395 | 3188 | 320207 | FALSE | TRUE |
| 272.11 | Hypercholesterolemia | endocrine/ metabolic | 1.018 | 0.018 | 0.005 | 1.008 | 1.029 | 4.78E-04 | 324759 | 47440 | 277319 | FALSE | TRUE |
| 411.4 | Coronary atherosclerosis | circulatory system | 1.023 | 0.023 | 0.007 | 1.010 | 1.037 | 5.27E-04 | 313406 | 26349 | 287057 | FALSE | TRUE |
| 272 | Disorders of lipoid metabolism | endocrine/ metabolic | 1.017 | 0.017 | 0.005 | 1.007 | 1.027 | 5.63E-04 | 328652 | 51333 | 277319 | FALSE | TRUE |
| 272.1 | Hyperlipidemia | endocrine/ metabolic | 1.017 | 0.017 | 0.005 | 1.007 | 1.027 | 6.45E-04 | 328449 | 51130 | 277319 | FALSE | TRUE |
| 585.1 | Acute renal failure | genitourinary | 1.031 | 0.031 | 0.009 | 1.013 | 1.050 | 9.30E-04 | 313931 | 12682 | 301249 | FALSE | TRUE |
| 244 | Hypothyroidism | endocrine/ metabolic | 1.024 | 0.024 | 0.007 | 1.009 | 1.039 | 1.12E-03 | 325419 | 21043 | 304376 | FALSE | TRUE |

Significance determined at FDR q<0.05. In addition Bonferroni threshold p<4.47 x 10-5

**Supplementary Table 10. Reverse causality MR assessment with CRP as outcome.**

| Exposure | Method | N SNPs* | Beta | SE | P value | heterogeneity | p_heterogeneity | intercept | p_pleiotropy | p_distortion |
|---|---|---|---|---|---|---|---|---|---|---|
| Schz | IVW | 95 | -0.004 | 0.003 | 0.160 | 513.354 | 5.05E-59 | | | |
| Schz | IVW-RE | 95 | -0.004 | 0.007 | 0.548 | 513.354 | 5.05E-59 | | | |
| Schz | MR-Egger | 95 | -0.012 | 0.031 | 0.702 | 512.999 | 2.47E-59 | 0.000614589 | 0.800 | |
| Schz | Weighted median | 95 | 1.26E-05 | 0.006 | 0.998 | | | | | |
| Schz | Weighted mode | 95 | 0.010 | 0.014 | 0.473 | | | | | |
| Schz | MR PRESSO | 81 | -0.006 | 0.005 | 0.227 | | | | | 0.776 |
| BC | IVW | 295 | 0.005 | 0.002 | 0.020 | 1319.647 | 1.31E-129 | | | |
| BC | IVW-RE | 295 | 0.005 | 0.005 | 0.272 | 1319.647 | 1.31E-129 | | | |
| BC | MR-Egger | 295 | 0.006 | 0.011 | 0.576 | 1319.628 | 6.21E-130 | -4.60E-05 | | |
| BC | Weighted median | 295 | 0.009 | 0.004 | 0.033 | | | | | |
| BC | Weighted mode | 295 | 0.012 | 0.005 | 0.028 | | | | | |
| BC | MR PRESSO | 278 | 0.005 | 0.003 | 0.096 | | | | | 0.975 |
| prca | IVW | 290 | 0.003 | 0.002 | 0.069 | 790.456 | 3.41E-48 | | | |
| prca | IVW-RE | 290 | 0.003 | 0.003 | 0.271 | 790.456 | 3.41E-48 | | | |
| prca | MR-Egger | 290 | 0.006 | 0.006 | 0.319 | 789.625 | 2.68E-48 | -0.000316886 | 0.582 | |
| prca | Weighted median | 290 | 0.006 | 0.003 | 0.049 | | | | | |
| prca | Weighted mode | 290 | 0.010 | 0.005 | 0.034 | | | | | |
| prca | MR PRESSO | 278 | 0.002 | 0.002 | 0.495 | | | | | 0.234 |
| COPD* | IVW | 13 | -0.004 | 0.004 | 0.293 | 52.870 | 4.34E-07 | | | |
| COPD | IVW-RE | 13 | -0.004 | 0.008 | 0.616 | 52.870 | 4.34E-07 | | | |
| COPD | MR-Egger | 13 | 0.021 | 0.026 | 0.428 | 48.156 | 1.34E-06 | -0.004922581 | 0.322 | |
| COPD | Weighted median | 13 | -0.006 | 0.006 | 0.362 | | | | | |
| COPD | Weighted mode | 13 | -0.005 | 0.009 | 0.595 | | | | | |
| COPD | MR PRESSO | 11 | -0.011 | 0.004 | 0.030 | | | | | 0.469 |

* COPD exposure variants selected at lowered threshold 1e-5 due to low number of genome wide significant SNPs harmonised with CRP summary statistics.

*N SNPs for MR PRESSO reported number of variants in MR after outlier correction

Significance determined by Bonferroni threshold $p < 0.0125$

p_pleiotropy= global p value from MR PRESSO, intercept p value from MR Egger

**Abbreviations**

"BC" = Breast Cancer

"PrC" = Prostate Cancer

"Schz" = Schizophrenia

"COPD"=Chronic Obstructive Pulmonary Disease

## Supplementary References


1. Watanabe, K., Taskesen, E., van Bochoven, A. & Posthuma, D. Functional mapping and annotation of genetic associations with FUMA. *Nat. Commun.* **8**, 1826 (2017).
2. Wang, K., Li, M. & Hakonarson, H. ANNOVAR: functional annotation of genetic variants from high-throughput sequencing data. *Nucleic Acids Res.* **38**, e164–e164 (2010).
3. Consortium*, T. Gte. The Genotype-Tissue Expression (GTEx) project. *Database Natl. Cent. Biomed. Inf.* **45**, 580–585 (2013).
4. Ramasamy, A. *et al.* Genetic variability in the regulation of gene expression in ten regions of the human brain. *Nat. Neurosci.* **17**, 1418–1428 (2014).
5. Fromer, M. *et al.* Gene expression elucidates functional impact of polygenic risk for schizophrenia. *Nature Neuroscience* vol. 19 1442–1453 (2016).
6. Ng, B. *et al.* An xQTL map integrates the genetic architecture of the human brain's transcriptome and epigenome. *Nat. Neurosci.* **20**, 1418–1426 (2017).
7. Grundberg, E. *et al.* Mapping cis-and trans-regulatory effects across multiple tissues in twins. *Nat. Genet.* **44**, 1084–1089 (2012).
8. Westra, H.-J. *et al.* Systematic identification of trans eQTLs as putative drivers of known disease associations. *Nat. Genet.* **45**, 1238–1243 (2013).
9. Võsa, U. *et al.* Unraveling the polygenic architecture of complex traits using blood eQTL metaanalysis. *bioRxiv* 447367 (2018) doi:10.1101/447367.
10. Schmiedel, B. J. *et al.* Impact of Genetic Polymorphisms on Human Immune Cell Gene Expression. *Cell* **175**, 1701-1715.e16 (2018).
11. Van Der Wijst, M. G. P. *et al.* Single-cell RNA sequencing identifies celltype-specific cis-eQTLs and co-expression QTLs. *Nat. Genet.* **50**, 493–497 (2018).
12. Wang, D. *et al.* Comprehensive functional genomic resource and integrative model for the human brain. *Science (80-. ).* **362**, (2018).
13. Schmitt, A. D. *et al.* A Compendium of Chromatin Contact Maps Reveals Spatially Active

Regions in the Human Genome. *Cell Rep.* **17**, 2042–2059 (2016).

14. Andersson, R. *et al.* An atlas of active enhancers across human cell types and tissues. *Nature* **507**, 455–461 (2014).

15. Forrest, A. R. R. *et al.* A promoter-level mammalian expression atlas. *Nature* **507**, 462–470 (2014).

16. Bertin, N. *et al.* Data Descriptor: Linking FANTOM5 CAGE peaks to annotations with CAGEscan. *Sci. Data* **4**, (2017).

17. de Leeuw, C. A., Mooij, J. M., Heskes, T. & Posthuma, D. MAGMA: Generalized Gene-Set Analysis of GWAS Data. *PLOS Comput. Biol.* **11**, e1004219 (2015).

18. Abdi, H. *The Bonferonni and Šidák Corrections for Multiple Comparisons*. *Encyclopedia of Measurement and Statistics* (SAGE Publications, 2007).

19. Pers, T. H. *et al.* Biological interpretation of genome-wide association studies using predicted gene functions. *Nat. Commun.* **6**, 5890 (2015).

20. Altshuler, D. L. *et al.* A map of human genome variation from population-scale sequencing. *Nature* **467**, 1061–1073 (2010).

21. Chang, C. C. *et al.* Second-generation PLINK: rising to the challenge of larger and richer datasets. **4**, 7 (2015).
