# Supplementary material for: Genetic analysis of over half a million people characterises C-reactive protein loci
Source: Nat Commun. 2022 Apr 22;13:2198. doi: 10.1038/s41467-022-29650-5 (PMC9033829; doi:10.1038/s41467-022-29650-5)
Supplement: Supplementary file 2 — Description of Additional Supplementary Files [file 41467_2022_29650_MOESM2_ESM.pdf]

Description of Additional Supplementary Files

File Name: Supplementary Data 1

Description: Genome-wide significant genomic loci associated to CRP.

File Name: Supplementary Data 2

Description: Credible set of the Bonferroni significant loci.

File Name: Supplementary Data 3

Description: Genes significantly associated to CRP associated genetic variants in gene based test using MAGMA.

File Name: Supplementary Data 4

Description: Genes implicated by mapping of SNPs across all four strategies in FUMA; positional, eQTL, chromatin mapping and MAGMA gene-based association analysis.

File Name: Supplementary Data 5

Description: Significant gene sets from DEPICT analysis.

File Name: Supplementary Data 6

Description: Cross-trait LDSC genetic correlation of CRP to previously published GWAS traits.

File Name: Supplementary Data 7

Description: MR-UKB association of CRP proxied genetic variables with 27 PheWAS outcomes.

File Name: Supplementary Data 8

Description: MR-replication results of CRP proxied genetic variables with 22 PheWAS identified outcomes extracted from MR-base database.

File Name: Supplementary Data 9

Description: MR association of CRP GWAS meta-analysis instrumental variants with eleven outcomes.

File Name: Supplementary Data 10

Description: MR association of CRP cis instrumental variants with eleven outcomes.
