# Supplementary material for: Genetic analysis of over half a million people characterises C-reactive protein loci
Source: Nat Commun. 2022 Apr 22;13:2198. doi: 10.1038/s41467-022-29650-5 (PMC9033829; doi:10.1038/s41467-022-29650-5)
Supplement: Supplementary file 13 — Reporting Summary [file 41467_2022_29650_MOESM13_ESM.pdf]

nature research

Corresponding author(s): Abbas Dehghan

Last updated by author(s): 18/02/2022

Reporting Summary

Nature Research wishes to improve the reproducibility of the work that we publish. This form provides structure for consistency and transparency in reporting. For further information on Nature Research policies, see our Editorial Policies and the Editorial Policy Checklist.

Statistics

For all statistical analyses, confirm that the following items are present in the figure legend, table legend, main text, or Methods section.

n/a	Confirmed	
[ ]	[x]	The exact sample size (*n*) for each experimental group/condition, given as a discrete number and unit of measurement
[x]	[ ]	A statement on whether measurements were taken from distinct samples or whether the same sample was measured repeatedly
[ ]	[x]	The statistical test(s) used AND whether they are one- or two-sided<br>*Only common tests should be described solely by name; describe more complex techniques in the Methods section.*
[ ]	[x]	A description of all covariates tested
[ ]	[x]	A description of any assumptions or corrections, such as tests of normality and adjustment for multiple comparisons
[ ]	[x]	A full description of the statistical parameters including central tendency (e.g. means) or other basic estimates (e.g. regression coefficient) AND variation (e.g. standard deviation) or associated estimates of uncertainty (e.g. confidence intervals)
[ ]	[x]	For null hypothesis testing, the test statistic (e.g. *F*, *t*, *r*) with confidence intervals, effect sizes, degrees of freedom and *P* value noted<br>*Give P values as exact values whenever suitable.*
[x]	[ ]	For Bayesian analysis, information on the choice of priors and Markov chain Monte Carlo settings
[x]	[ ]	For hierarchical and complex designs, identification of the appropriate level for tests and full reporting of outcomes
[ ]	[x]	Estimates of effect sizes (e.g. Cohen's *d*, Pearson's *r*), indicating how they were calculated

Our web collection on statistics for biologists contains articles on many of the points above.

Software and code

Policy information about availability of computer code

Data collection	No software was used for data collection
Data analysis	BOLT-LMM v.2.3 , R v.3.5.3 ,FUMA v1.3.5d, DEPICT v1rel1 94, MAGMA v1.6, PheWAS v 0.99.5-3, TwoSampleMR v0.4.26 , LD-hub v1.9.0, METAL version released on 2011-03-25, PLINK v1.9.

For manuscripts utilizing custom algorithms or software that are central to the research but not yet described in published literature, software must be made available to editors and reviewers. We strongly encourage code deposition in a community repository (e.g. GitHub). See the Nature Research guidelines for submitting code & software for further information.

Data

Policy information about availability of data

All manuscripts must include a data availability statement. This statement should provide the following information, where applicable:

- Accession codes, unique identifiers, or web links for publicly available datasets
- A list of figures that have associated raw data
- A description of any restrictions on data availability

UK Biobank application ID13436. Summary statistics of the CHARGE CRP GWAS used in this study is publicly available from the GWAS catalogue accession code GCST007615 (https://www.ebi.ac.uk/gwas/). The derived CRP GWAS meta-analysis summary statistics generated in this study has been deposited in the GWAS catalogue under accession code GCST00186 (https://www.ebi.ac.uk/gwas/). Human genome assembly GRCh37 (hg19) from Genome Reference Consortium (https://www.sanger.ac.uk/data/genome-reference-consortium/).

Field-specific reporting

Life sciences study design

All studies must disclose on these points even when the disclosure is negative.

Sample size	Total 575,531. This sample size was determined by the participant data from UK Biobank post exclusions and CHARGE summary statistics.
Data exclusions	Predetermined exclusion of non white participants, participants on immune modulating drugs and those who had auto-immune conditions.
Replication	Replicated previously reported loci summary statistics with the meta-analysis summary statistics once.
Randomization	N/A. We did not use any study design that required randomisation.
Blinding	N/A. We did not use any study design that required blinding.

Reporting for specific materials, systems and methods

We require information from authors about some types of materials, experimental systems and methods used in many studies. Here, indicate whether each material, system or method listed is relevant to your study. If you are not sure if a list item applies to your research, read the appropriate section before selecting a response.

Materials & experimental systems

n/a	Involved in the study	
☒	☐	Antibodies
☒	☐	Eukaryotic cell lines
☒	☐	Palaeontology and archaeology
☒	☐	Animals and other organisms
☐	☒	Human research participants
☒	☐	Clinical data
☒	☐	Dual use research of concern

Methods

n/a	Involved in the study	
☒	☐	ChIP-seq
☒	☐	Flow cytometry
☒	☐	MRI-based neuroimaging

Human research participants

Policy information about studies involving human research participants

Population characteristics	54% females, mean age 57, BMI 27.4, CRP 2.6 mg/l
Recruitment	UK Biobank recruitment of participants.
Ethics oversight	North West Multi-Centre Research Ethics Committee (11/NW/0382)

Note that full information on the approval of the study protocol must also be provided in the manuscript.
